# Lactylome Analysis Unveils Lactylation‐Dependent Mechanisms of Stemness Remodeling in the Liver Cancer Stem Cells

**DOI:** 10.1002/advs.202405975

**Published:** 2024-08-05

**Authors:** Fan Feng, Jiaqin Wu, Qingjia Chi, Shunshun Wang, Wanqian Liu, Li Yang, Guanbin Song, Lianhong Pan, Kang Xu, Chunli Wang

**Affiliations:** ^1^ Hubei Shizhen Laboratory Wuhan 430065 China; ^2^ School of Pharmacy Hubei University of Chinese Medicine Wuhan 430065 China; ^3^ School of Laboratory Medicine Hubei University of Chinese Medicine Wuhan 430065 China; ^4^ National Innovation and Attracting Talents “111” base Key Laboratory of Biorheological Science and Technology Ministry of Education College of Bioengineering Chongqing University Chongqing 400000 China; ^5^ Department of Engineering Structure and Mechanics School of Science Wuhan University of Technology Wuhan 430070 China; ^6^ Chongqing Key Laboratory of Development and Utilization of Genuine Medicinal Materials in Three Gorges Reservoir Area Chongqing Engineering Research Center of Antitumor Natural Drugs Chongqing Three Gorges Medical College Chongqing 400030 China; ^7^ Center of Traditional Chinese Medicine Modernization for Liver Diseases Hubei University of Chinese Medicine Wuhan 430065 China

**Keywords:** glycolysis, lactylation, lactylome, liver cancer stem cells, stemness

## Abstract

Lactate plays a critical role as an energy substrate, metabolite, and signaling molecule in hepatocellular carcinoma (HCC). Intracellular lactate‐derived protein lysine lactylation (Kla) is identified as a contributor to the progression of HCC. Liver cancer stem cells (LCSCs) are believed to be the root cause of phenotypic and functional heterogeneity in HCC. However, the impact of Kla on the biological processes of LCSCs remains poorly understood. Here enhanced glycolytic metabolism, lactate accumulation, and elevated levels of lactylation are observed in LCSCs compared to HCC cells. H3K56la was found to be closely associated with tumourigenesis and stemness of LCSCs. Notably, a comprehensive examination of the lactylome and proteome of LCSCs and HCC cells identified the ALDOA K230/322 lactylation, which plays a critical role in promoting the stemness of LCSCs. Furthermore, this study demonstrated the tight binding between aldolase A (ALDOA) and dead box deconjugate enzyme 17 (DDX17), which is attenuated by ALDOA lactylation, ultimately enhancing the regulatory function of DDX17 in maintaining the stemness of LCSCs. This investigation highlights the significance of Kla in modulating the stemness of LCSCs and its impact on the progression of HCC. Targeting lactylation in LCSCs may offer a promising therapeutic approach for treating HCC.

## Graphical abstract

The study demonstrates the significant impact of lactylation on the maintenance of stemness in LCSCs, highlighting the crucial role of lactate‐derived H3 histone and ALDOA K230/322 lactylation in initiating the transcription of genes related to stemness. These findings suggest that lactate directly influences the proliferation, migration, and stemness properties of LCSCs through lactylation, offering a promising therapeutic strategy for HCC.

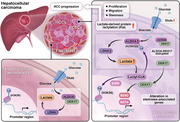

## 1 Introduction

Hepatocellular carcinoma (HCC), the predominant histological subtype of primary liver cancer, ranks as the third leading cause of cancer‐related mortality globally.^[^ ^1^, ^2^, ^3^ ^]^ HCC exhibits significant cellular heterogeneity, organized hierarchically, with distinct subpopulations displaying varying functional roles in pathophysiology.^[^ ^4^ ^]^ Extensive research has established a correlation between high heterogeneity in HCC and unfavorable clinical prognosis.^[^ ^5^, ^6^ ^]^ The existence of liver cancer stem cells (LCSCs) laboring self‐renewal and differentiation characteristics partially explains HCC heterogeneity.^[^ ^7^, ^8^ ^]^ LCSCs play a crucial role in tumor development and resistance to treatment. Understanding their fate within a tumor could lead to new therapeutic options.^[^ ^9^ ^]^

Aerobic glycolysis is a key feature of cancer metabolism and allows tumor cells to cope with the increased energy demands of their high proliferation rate.^[^ ^10^, ^11^ ^]^ Lactate, a byproduct of glycolysis metabolism, has been demonstrated to exert a significant influence on tumor progression, metastasis, and immune evasion through diverse mechanisms.^[^ ^12^ ^]^ Within the highly heterogeneous tumor microenvironment, the impact of lactate on cells with varying metabolic characteristics and phenotypes can be intricate and hard to decipher.^[^ ^13^ ^]^ Lactate plays a dual role within the tumor microenvironment, serving as both a supportive energy source for activated immune cells and a signaling molecule that hinders immune cell cytotoxicity.^[^ ^14^ ^]^ Our research group has observed fluctuations in lactate levels within LCSCs that are implicated in the progression of HCC,^[^ ^15^ ^]^ yet the underlying molecular mechanisms remain incompletely understood.

A recent study has identified a novel function for lactate as a substrate for a newly discovered posttranslational modification, histone lysine lactylation (Kla), which directly regulates gene transcription.^[^ ^16^, ^17^ ^]^ Increasing evidences support the critical roles of histone Kla in promoting oncogenesis and immunosuppression.^[^ ^18^, ^19^ ^]^ Protein Kla has been detected in HCC tumors and is linked to the progression of HCC.^[^ ^20^, ^21^ ^]^ The discovery of histone Kla marks and their dynamic changes suggest a potential role in the tumorigenicity of LCSCs.^[^ ^15^ ^]^ Meanwhile, interfering with Kla modifiers through the use of small‐molecule inhibitors, such as targeting lactate dehydrogenase‐A (LDHA), has been shown to elicit the potent antitumor responses.^[^ ^22^, ^23^, ^24^ ^]^ These results prompt further scientific inquiry into the potential role of protein Kla in regulating the malignant biological behavior of LCSCs. Further investigation is warranted to elucidate the precise regulatory mechanisms by which protein Kla modifications control the function of LCSCs.

In this study, we have demonstrated a significant increase in lactylations in HCC, particularly in LCSCs. Our screening results indicate that H3K56la plays a crucial role in the tumorigenesis and maintenance of stemness in LCSCs by regulating target genes. Interestingly, we have observed a marked rise in Kla levels on non‐histone proteins in LCSCs, with a focus on aldolase A (ALDOA) K230/322 lactylation contributing to the maintaining the stemness of these cells. The increased ALDOA K230/322 levels disrupt interaction of ALDOA and dead box deconjugate enzyme 17 (DDX17), leading to alterations in genes associated with stemness. These novel findings have the potential to advance our understanding of HCC progression.

## 2 Results

### 2.1 LCSCs Exhibit a Higher Level of Lactylation Compared to HCC Cells

**Figure 1:**
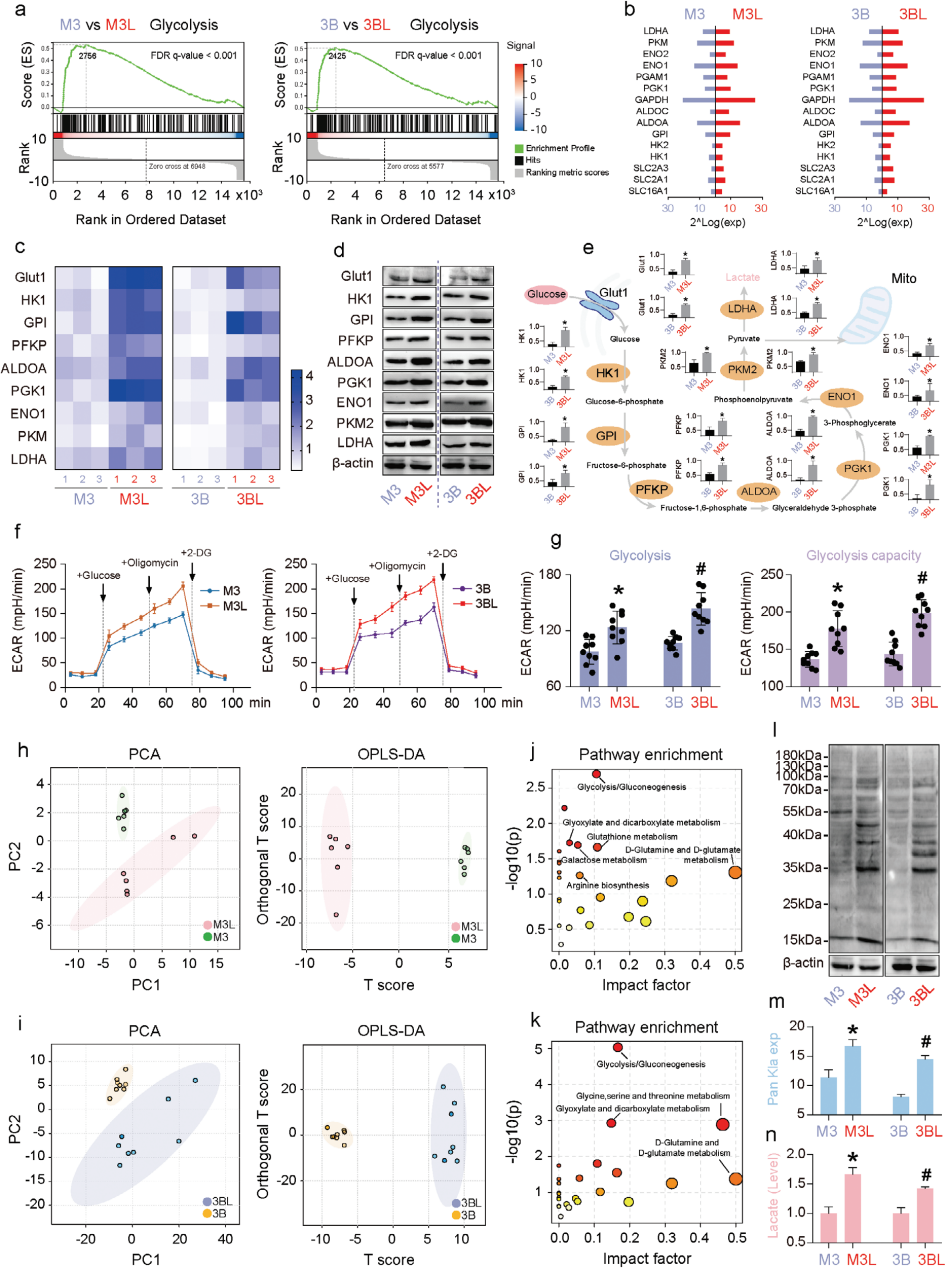
LCSCs display higher glycolytic metabolism, lactate accumulation, and lactylation level than HCC cells. a) GSEA analyses based on the RNA‐seq data from M3 (HCCLM3) versus M3‐based LCSCs (M3L) and 3B (Hep3B) versus 3 B‐based LCSCs (3BL); b) Glycolytic‐related gene expression from RNA‐seq data; c) qRT‐PCR detection of the core glycolytic gene expressions; d) Western blotting analysis of the core glycolytic protein expressions, e) the statistical analyses based on (d); f) The ECAR analyses of M3 versus M3L and 3B versus 3BL, g) the statistical analyses based on (f); h) PCA and OPLS‐DA analyses of metabonomics of M3 versus M3L, j) Pathway enrichment based on the differential metabolites of M3 versus M3L; i) PCA and OPLS‐DA analyses of metabonomics of 3B versus 3BL, k) Pathway enrichment based on the differential metabolites of 3B versus 3BL; l) Pan‐Kla (Pan lysine lactylation) of LCSCs versus HCC cells, m) the statistical analyses based on (l); n) Intracellular lactate production measurements of LCSCs and HCC cells. (*) and (#) *p* < 0.05 indicates significant difference.

Our results showed that LCSCs enriched from HCC cells had greater self‐renewal and proliferative capacity, higher expression of stemness markers, and greater tumorigenicity (Figure S1, Supporting Information). Next, transcriptome sequencing was conducted on HCC cells and enriched LCSCs. GSEA enrichment analysis of differentially expressed genes revealed significant differences in glycolytic pathways between M3 and M3L, and 3B and 3BL (**Figure** **1**a). Transcriptome sequencing revealed that the mRNA expression levels of glycolytic enzymes, including GLUT1, HK1, GPI, PFKP, ALDOA, PGK1, PKM2, and LDHA, were significantly upregulated in LCSCs compared with HCC cells (Figure 1b). This result was verified through qRT‐PCR and western blotting assays (Figure 1c–e). The extracellular acidification rate (ECAR) difference between HCC cells and LCSCs was measured using the extracellular flux detector Seahorse XF. The results indicate that LCSCs have a higher level of glycolysis level and glycolysis capacity compared to HCC cells (Figure 1f,g). The metabolic differences between HCC cells and LCSCs were compared using GC‐MS metabolomics (Figure 1h,i). The results showed that differential metabolites were enriched in glycolysis/gluconeogenesis (Figure 1j,k). The western blotting analysis of the Pan Kla antibody revealed higher levels of lactylation expression in LCSCs when compared to HCC cells (Figure 1l,m). Both metabolomics and lactate content assay experiments have shown that the intracellular lactate content of LCSCs is significantly higher than that of HCC cells (Figure 1n). These results suggest that LCSCs exhibit higher levels of glycolysis compared to HCC cells. These lead to an increase in intracellular lactate, which in turn provides a substrate for intracellular lactylation in LCSCs.

### 2.2 Altering the Level of Lactylation Affects the Proliferative Capacity and Stem Cell Properties of LCSCs

In order to investigate the role of lactylation in HCC, we first compared the expression of the lactylation pan antibody Pan Kla in 90 groups of HCC tissues and the corresponding 90 groups of paraneoplastic normal tissues by tissue microarray technology and tissue immunofluorescence. Based on the fluorescence intensity statistics, the results showed that the overall lactylation level of HCC tumor tissues was significantly higher than that of paracancerous normal tissues (Figure S2a–c, Supporting Information). The fluorescence intensity of immunofluorescence staining of different stages of HCC was further compared according to the AJCCA clinical stage of HCC, and the results showed that the fluorescence intensity of HCC stage 2 was significantly higher than that of HCC stage 1, suggesting that the level of lactylation increased with the development of HCC (Figure S2d, Supporting Information). Studies have shown that the distribution of CSCs in cancerous tissues is not homogeneous, and CSCs are more likely to be distributed in the invasive front region of the tumor than in the core region of the tumor.^[^ ^25^ ^]^ Immunofluorescence staining of HCC tissues showed higher expression of stemness markers CD44, OCT4, and Kla in the invasion front region compared to the core region of HCC tissues, suggesting that the level of lactylation in the invasion front region where LCSCs were located was significantly higher than that in the core region of the HCC cancer tissues (Figure S2e,f, Supporting Information).

**Figure 2:**
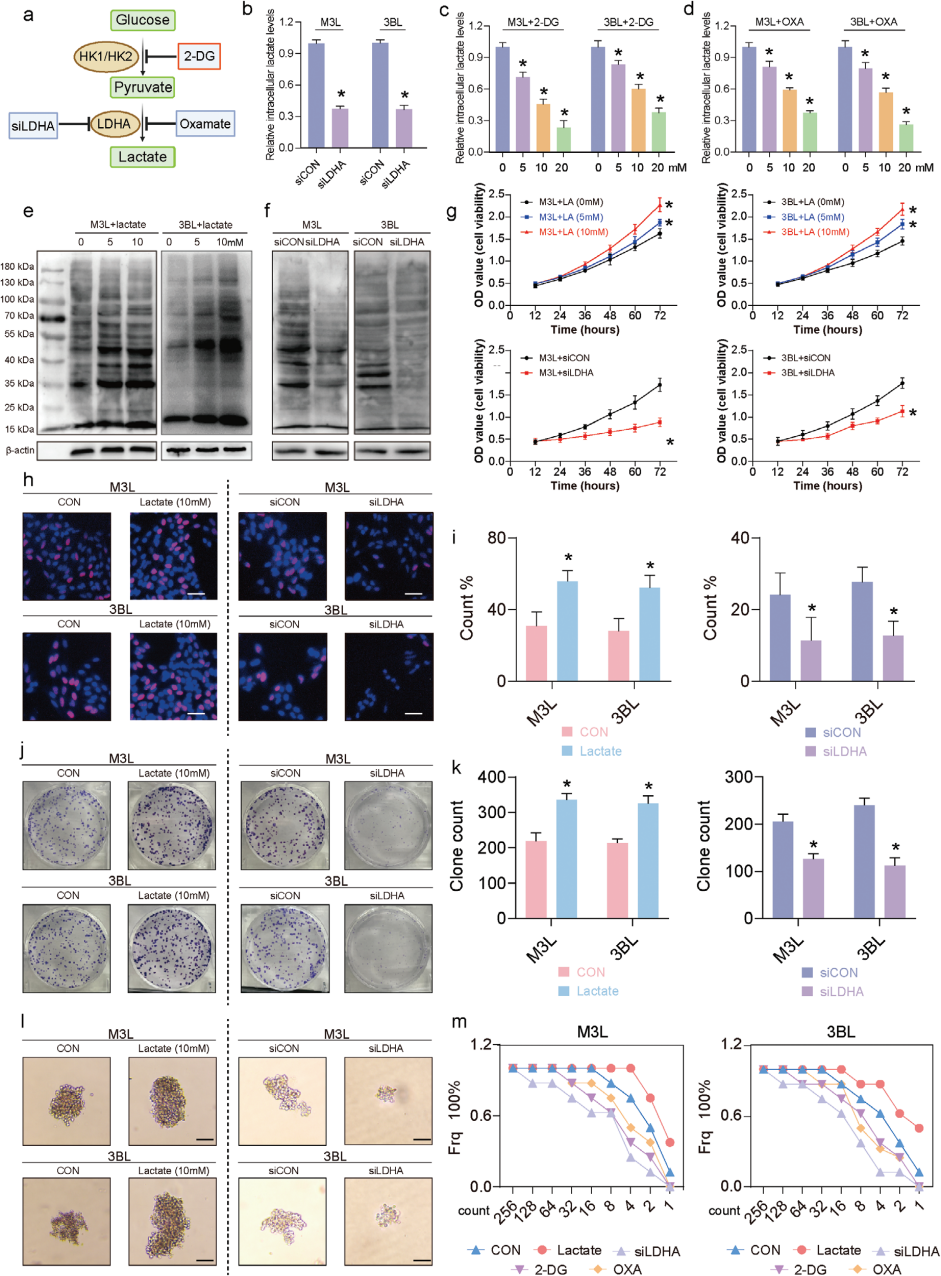
Altering the level of lactylation affects the tumorigenesis capacity and stemness properties of LCSCs. a) Experimental procedure of glycolytic inhibition; b–d) The intracellular lactate levels of M3L and 3BL with (b) LDHA silence (siLDHA), (c) 2‐DG treatment, (d) Oxamate (OXA) treatment; e) The Pan‐Kla detection of M3L and 3BL with different treatments; f,g) The cell viabilities of M3L and 3BL with (f) LA (lactate) treatments and with (g) LDHA silence (siLDHA); h) Cell proliferation from EdU assay of M3L and 3BL with different treatments (scale bar = 50 µm), i) the statistical analyses of (h); j) Colony formation abilities of M3L and 3BL with different treatments, k) the statistical analyses of (j); l) Sphere formation abilities of M3L and 3BL with different treatments (scale bar = 200 µm); m) M3L and 3BL frequency with different treatments were determined using in vitro LDA (Limiting dilution analysis); (*) *p* < 0.05 indicates significant difference.

To further assess whether lactylation of LCSCs is associated with the development of HCC, we altered the overall intracellular lactylation level in LCSCs by adding lactate or inhibiting lactate production (**Figure** **2**a). Treatment of LCSCs with siRNA for lactate dehydrogenase A (LDHA) and glycolysis inhibitors (glucose analogue 2‐deoxy‐d‐glucose (2‐DG) and Oxamate) significantly reduced the intracellular lactate levels in LCSCs, and the higher the concentration of 2‐DG and Oxamate used, the lower the lactate levels were (Figure 2b–d). Pan Kla's western blotting and flow cytometry results showed that exogenous lactate (5 and 10 mm) treatments significantly increased the overall lactylation level of LCSCs, and the extent of the rise was proportional to the exogenous lactate concentration (Figure 2e; Figure S2g,h, Supporting Information). In contrast, siLDHA and glycolysis inhibitor treatments significantly suppressed the overall lactylation level of LCSCs (Figure 2f; Figure S2i–k, Supporting Information). Next, we examined the effects of altered lactylation on the biological behaviors of LCSCs. The results showed that exogenous lactate addition significantly promoted the proliferative capacity of LCSCs, whereas siLDHA and glycolysis inhibitor treatments significantly inhibited the proliferative capacity of LCSCs (Figure 2g; Figure S3a, Supporting Information). Edu proliferation staining assay showed a significant increase in the number of positive cells for LCSC in the group treated with the addition of exogenous lactate and a significant decrease in the number of positive cells for EdU in the group treated with siLDHA and glycolysis inhibitors (Figure 2h,i; Figure S3b, Supporting Information). The results of the clone formation assay were in agreement with the EdU assay (Figure 2j,k; Figure S3c, Supporting Information). Flow cytometry revealed that 10 mm exogenous lactate treatment increased the number of LCSCs entering S phase from 26.36% to 33.79% for M3L and from 26.35% to 30.84% for 3BL. Whereas siLDHA and glycolysis inhibitor treatments decreased the number of cells of LCSCs entering S phase. These results above suggest that regulating the level of lactylation in LCSCs by altering glycolysis level can modulate their cell cycle (Figure S3d, Supporting Information). The results of transwell experiments showed that exogenous lactate addition significantly enhanced the migration and invasion abilities of LCSCs, whereas reduction of lactate production in LCSCs by siLDHA and glycolysis inhibitor treatment significantly inhibited the migration and invasion abilities of LCSCs (Figure S3e,f, Supporting Information).We next further examined the effect of altered lactylations on the stem cell properties of LCSCs and showed that 10 mm lactate significantly increased the size of tumor cell spheroids formed by LCSCs as well as the stem cell frequency from 1:3.4 to 1:1.4 and from 1:5.0 to 1:2.1 (Figure 2l,m). In contrast, siLDHA and glycolysis inhibitor treatment significantly suppressed the size of tumor cell spheroids formed by LCSCs as well as the stem cell frequency (Figure S4a,b, Supporting Information). RT‐qPCR and western blotting results showed that exogenous lactate significantly promoted the mRNA and protein expression of CD44, CD90, CD133, SOX2, Nanog, and OCT4 in LCSCs compared with controls. In contrast, siLDHA as well as the glycolysis inhibitors 2‐DG and oxamate significantly inhibited mRNA and protein expression of LCSCs stemness markers (Figure S4c–e, Supporting Information). These results suggest that lactylation plays an important role in regulating the proliferative capacity and stem cell properties of LCSCs.

### 2.3 H3K56la is the Major Histone Lactylation Site in LCSCs

**Figure 3:**
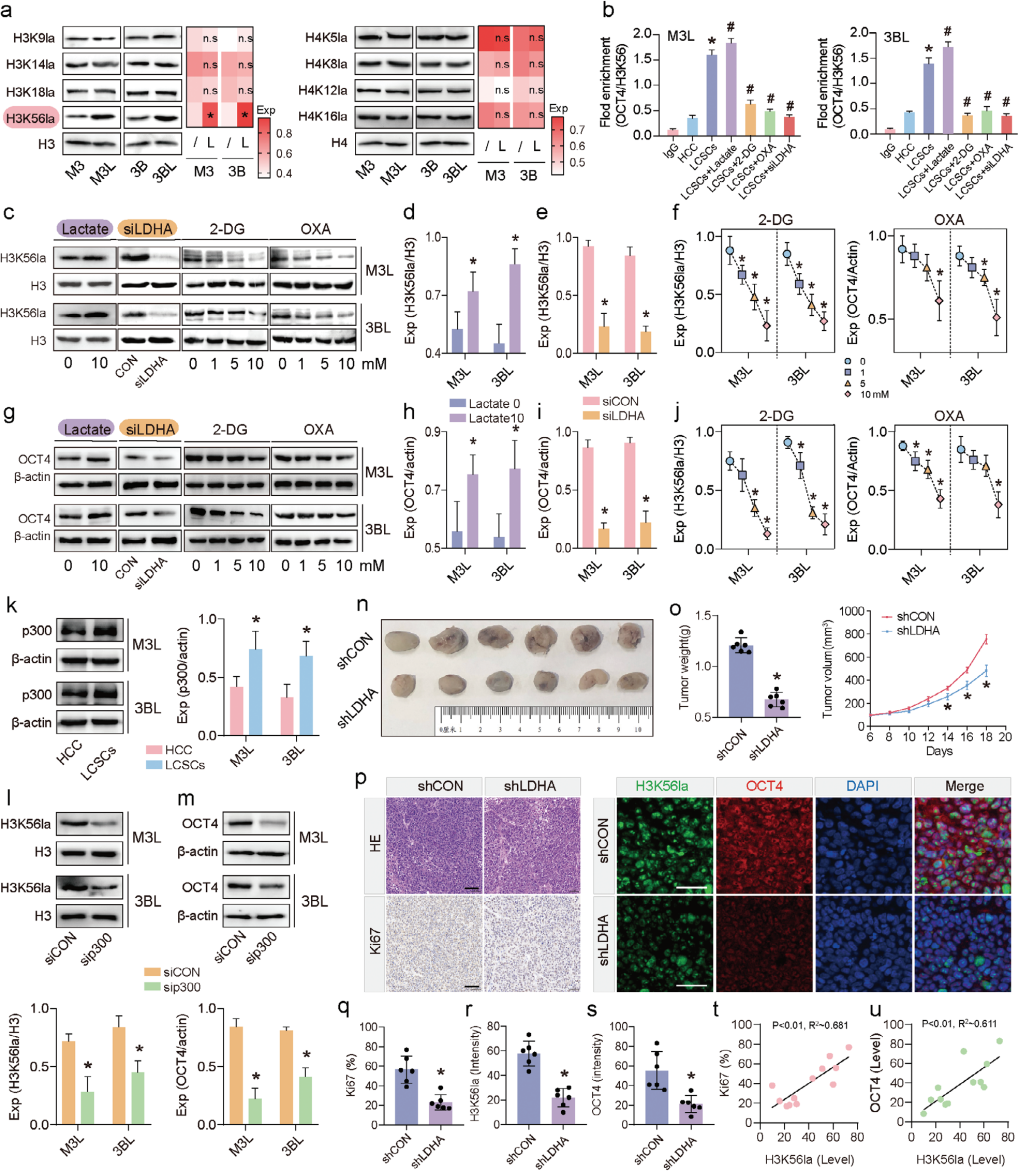
H3K56la is the major histone lactylation site in LCSCs. a) The detection of histone lactylations with western blotting, H3K56la site was significantly (*) increased in LCSCs versus HCC cells; b) ChIP‐PCR analyses of OCT4 via reacting with immunoprecipitation of H3K56la antibody in LCSCs with different treatments(^*^ *p* < 0.05, compared to HCC cells) (^#^ *p* < 0.05, compared to LCSCs); c–f) The detection of H3K56la with western blotting in M3L and 3BL with different treatments, d–f) The statistical analyses of (c); g–j) The detection of OCT4 with western blotting in M3L and 3BL with different treatments, h–j) the statistical analyses of (g); k) The detection of p300 with western blotting in LCSCs and HCC cells; l) The detection of H3K56la with western blotting in LCSCs with p300 silence (sip300); m) The detection of OCT4 with western blotting in LCSCs with p300 silence (sip300); n,o) In vivo tumor formation of LCSCs with shLDHA, (o) Tumor weight and volume measurement; p) Histological and immunostaining evaluation of in vivo tumor tissues (scale bar = 100 µm); q) In vivo Ki67 intensity statistical analyses; s) In vivo H3K56la intensity statistical analyses; t) In vivo OCT4 intensity statistical analyses; u) The correlation analysis of H3K56la level and Ki67 count; v) The correlation analysis of H3K56la and OCT4 level. (*) p < 0.05 indicates significant difference, (n.s.) indicates non‐significant difference.

Further observation of western blotting results of HCC cells and LCSCs lactylated pan antibody revealed that the lactylation was found to be near 17 kDa (Figure 1l), suggesting that the LCSCs histones may have undergone a lactylation change. Therefore, in order to investigate whether these changes were indeed caused by the modification sites of histones, lactylation antibodies against histone H3 sites H3K9la, H3K14la, H3K18la, H3K56la and H4 sites H4K5la, H4K8la, H4K12la, H4K16la were further used to detect whether these sites were changed. The results showed that the expression levels of H3K56la were significantly higher in LCSCs compared to both HCCLM3 cells and Hep3B cells (**Figure** **3**a). Next, we used chromatin immunoprecipitation (ChIP‐PCR) to detect the binding of H3K56la to SOX2, Nanog, and OCT4 promoter regions in LCSCs. The results showed that there was no significant difference in the binding of H3K56la to SOX2 and Nanog promoter regions in HCC cells versus LCSCs, nor was there any significant change in the binding of H3K56la to SOX2 and Nanog promoter regions in LCSCs after treatment with lactate, siLDHA, and glycolysis inhibitors (Figure S4f, Supporting Information). However, the binding of H3K56la and OCT4 promoter regions was significantly elevated in LCSCs compared with HCC cells after exogenous lactate treatment. In contrast, the binding of H3K56la and OCT4 promoter region was significantly decreased in LCSCs after treatment with siLDHA as well as glycolysis inhibitors (Figure 3b). Western blotting experiments showed that the protein expression levels of H3K56la and OCT4 were significantly increased in LCSCs after treatment with exogenous lactate, whereas they were significantly decreased in LCSCs after treatment with siLDHA as well as glycolysis inhibitors (Figure 3c–j). Meanwhile, we observed that exogenous lactate addition did not alter the acetylation of histones H3K9, H3K14, H3K18, and H3K56 (Figure S4g, Supporting Information). These results suggest that LCSCs can promote OCT4 expression through the binding of H3K56la to the promoter region of the stemness marker OCT4.

The results showed a significant increase in p300 protein expression levels in LCSCs compared to HCC cells (Figure 3k). Treatment with sip300 significantly suppressed the expression of H3K56la and OCT4 in LCSCs. These results suggest that p300 may act as a lactoyltransferase of H3K56la in LCSCs (Figure 3l,m). The impact of lactylation changes on the tumorigenic ability of LCSCs was further investigated. The results indicate that LCSCs treated with shLDHA exhibited a significant reduction in tumor volume and weight compared to untreated LCSCs (Figure 3n,o). The H&E and Ki67 immunohistochemistry results showed a decrease in the number of proliferating cells in the shLDHA‐treated group compared to untreated LCSCs (Figure 3p,q). Immunofluorescence staining of H3K56la and OCT revealed that siLDHA treatment significantly inhibited the expression of H3K56la and OCT4 in tumors in vivo (Figure 3r,s). Correlation analysis showed that the expression of H3K56la in LCSCs in vivo was positively correlated with the expression of Ki67 and OCT4 (Figure 3t,u). These results suggest that the stronger tumorigenic ability of LCSCs may result from enhanced lactylation of the H3K56 locus.

### 2.4 LCSCs have a Higher Level of ALDOA Lactylation Level than HCC Cells

**Figure 4:**
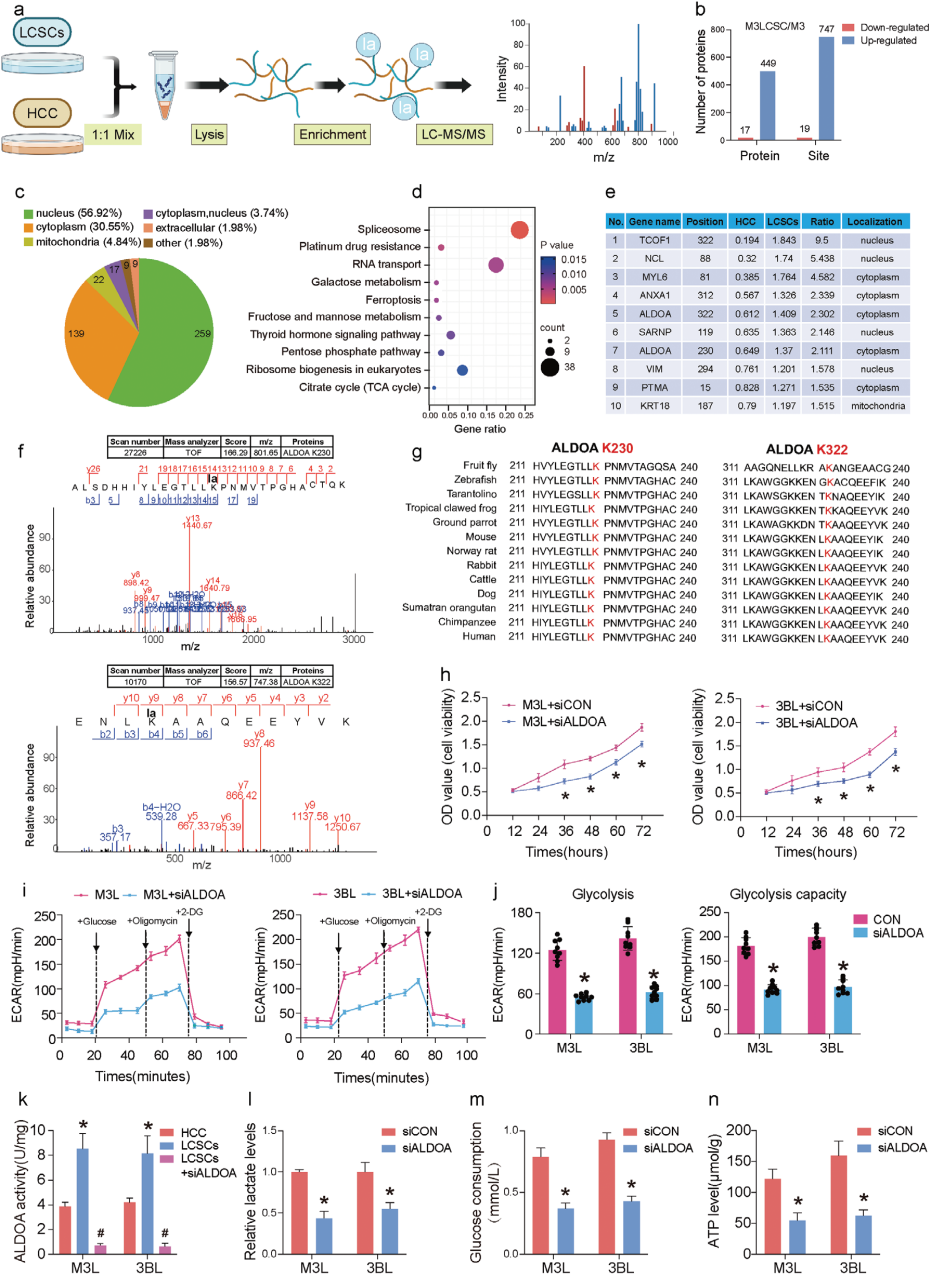
Global view of lactylated proteins in LCSCs compared to HCC cells. a) Flowchart of the lactylome; b) Differential lactylation proteins and loci of M3 and M3L; c) Subcellular localization of lactylated differential proteins; d) KEGG enrichment of lactylated differential proteins; e) Top ten differential multiples of lactylated differential proteins; f) Mass spectrometry of the ALDOA K230 and K322 loci; g) ALDOA K230 and K322 loci conserved; h) The cell viabilities of M3L and 3BL with siALDOA treatment; i) The ECAR analyses of M3L and 3BL with siALDOA, j) The statistical analyses based on (i); k) ALDOA enzyme activity assay; l) Lactate content assay; m) Glucose content assay; n) Pyruvate content assay; (*) *p* < 0.05 indicates significant difference.

We observed differences in the expression of the non‐histone region Pan Kla, in addition to histone 17 kDa, between HCC cells and LCSCs. This suggests that there are many non‐histone proteins in LCSCs that can also undergo lactylation. Next, HCCLM3 and M3L were collected for lactylome to compare lactylation of non‐histone proteins (**Figure** **4**a). The results showed that 466 proteins and 766 modification sites were altered in LCSCs with a threshold of 1.5‐fold or more compared to HCC cells (Figure S5a, Supporting Information). Among the identified proteins, 449 were found to have lactylation that were upregulated, while 17 proteins had downregulated lactylation. Additionally, 747 sites with lactylation were identified as upregulated and 19 as downregulated (Figure 4b). Quality control analysis showed that the peptide distribution was within reasonable limits (Figure S5b, Supporting Information). The differentially modified proteins (466 in total) were classified based on their subcellular structure annotation using WolF Psort software. The majority of these proteins were found in the nucleus, followed by the cytoplasm (Figure 4c). The analysis of Gene Ontology (GO) enrichment and KEGG pathway revealed that the differentially modified proteins were closely associated with cellular metabolic pathways, such as fructose and mannose metabolism, pentose phosphate pathway, and tricarboxylic acid (TCA) cycle (Figure 4d; Figure S5c–g, Supporting Information). The relative quantification of the top 10 proteins with increased lactylation is shown in Figure 4e. Differences in lactylation of both K322 and K230 of aldolase A (ALDOA), the fourth enzyme in glycolysis, were observed. Mass spectrometry data extracted from the raw data confirmed the presence of lactylation at these two sites (Figure 4f). The amino acid sequences of ALDOA from various species were examined on the Uniprot website (https://www.uniprot.org/). The conservation of the ALDOA K230 and K322 sites across a broad range of animals, from fruit fly and zebrafish to chimpanzees and human, suggests that these sites have been relatively stable throughout evolution and may have significant structures and functions (Figure 4g).

The analysis of the TCGA database revealed that ALDOA exhibits high expression in various tumor tissues, including HCC (Figure S6a,b, Supporting Information). Additionally, ALDOA expression tends to increase with the progression of HCC stage, indicating a potential association between ALDOA expression level and HCC progression (Figure S6c, Supporting Information). The Kaplan‐Meier survival curves indicate that patients with HCC and high ALDOA expression have a poor prognosis, with lower survival rates (Figure S6d, Supporting Information). The GSEA enrichment analysis revealed a significant association between high expression of ALDOA and HCC stemness, indicating that ALDOA plays a crucial role in the development of HCC (Figure S6e, Supporting Information). To investigate the function of highly expressed ALDOA in LCSCs, we knocked down its expression using siRNA for further study. To verify the success of siALDOA, we used western blotting to confirm the protein expression changes of ALDOA in LCSCs. The results showed that both siALDOA1 and siALDOA2 significantly reduced the protein expression of ALDOA in LCSCs compared to the positive control group. The inhibition effect was better in the siALDOA1 group, and therefore, we selected siALDOA1 for subsequent experiments (Figure S6f, Supporting Information). Subsequent to CCK8, clone formation, and EdU proliferation staining assays, it was observed that the proliferation ability of LCSCs was inhibited by ALDOA knockdown (Figure 4h; Figure S6g,h, Supporting Information). The results of transwell experiments showed that siALDOA inhibited the migration and invasion abilities of LCSCs (Figure S6i,j, Supporting Information). The Seahorse XF extracellular flow detector was used to measure the extracellular acidification rate (ECAR) of LCSCs, and the results showed that siALDOA treatment could significantly reduce the ECAR of LCSCs (Figure 4i,j). Meanwhile, our results also indicated that the enzymatic activity of ALDOA and key indicators of glycolysis, such as glucose consumption, intracellular lactate production, and ATP production, were significantly reduced in LCSCs after siALDOA treatment (Figure 4k–n). The above results indicated that siALDOA treatment could significantly reduce the glycolysis level of LCSCs.

### 2.5 ALDOA K230/322R Mutation Significantly Inhibits Proliferation Ability and Glycolysis Level in LCSCs

**Figure 5:**
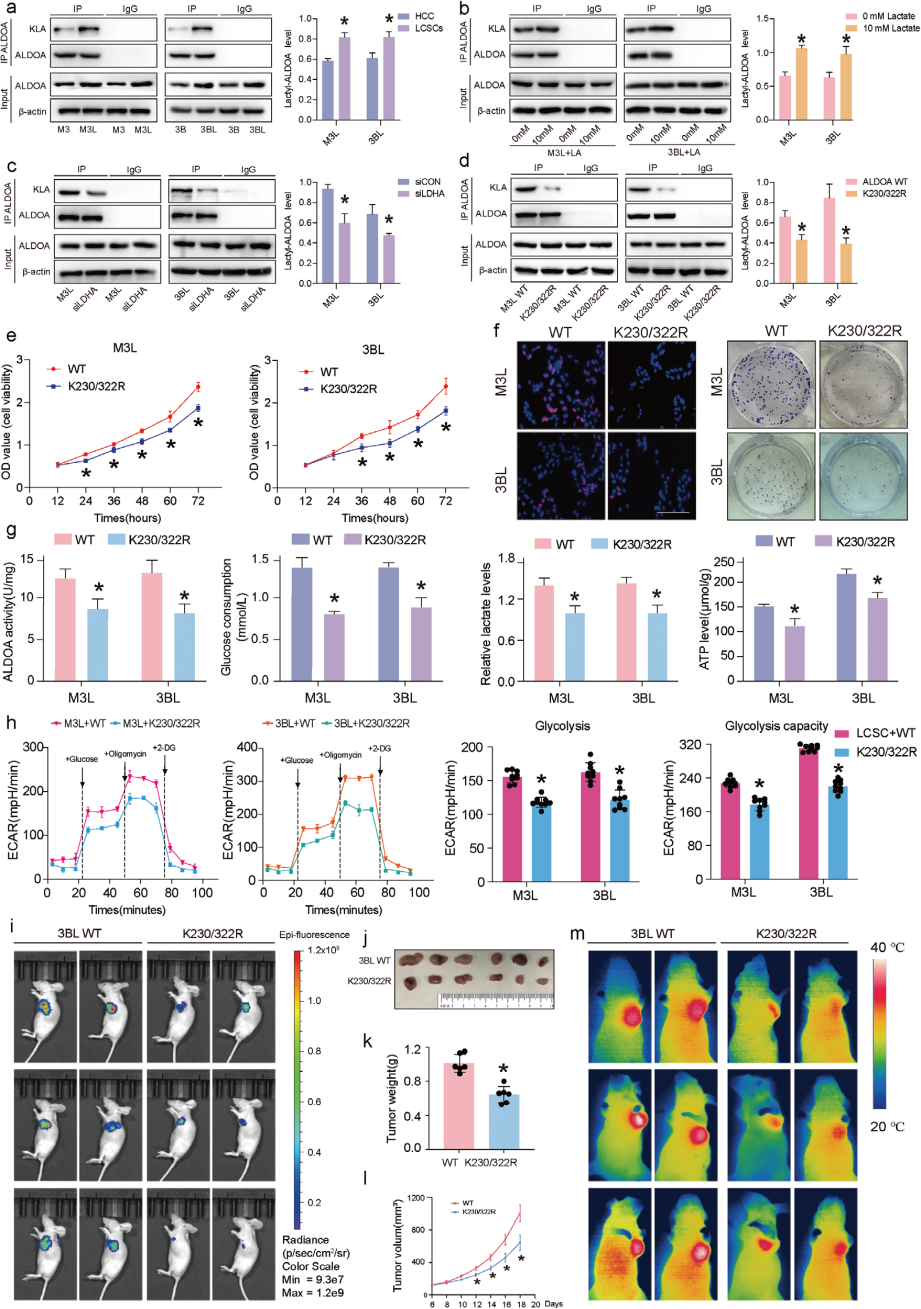
ALDOA K230/322R mutation significantly constrains tumorigenesis ability and glycolysis in LCSCs. a) Immunoprecipitation (IP) validation of ALDOA lactylation differences in HCC cells and LCSCs; b) The detection of ALDOA lactylation with IP in M3L and 3BL with lactate treatment; c) The detection of ALDOA lactylation with IP in M3L and 3BL with siLDHA treatment; d) The detection of ALDOA lactylation with IP in M3L and 3BL with ALDOA K230/322R mutation treatment; e) The cell viabilities of M3L and 3BL with ALDOA K230/322R mutation treatment; f) Cell proliferation from EdU assay and colony formation of M3L and 3BL with ALDOA K230/322R mutation treatment (scale bar = 100 µm); g) ALDOA activity, lactate content assay, glucose content assay and pyruvate content assay; h) The ECAR analyses of M3L and 3BL with ALDOA K230/322R mutation treatment; i) Live imaging of nude mice injected with 3BL WT and 3BL K230/322R mutation; j–m) In vivo tumor formation of LCSCs with ALDOA K230/322R mutation, (j) tumor imaging; (k) Tumor weight measurement, (l) Tumor volume measurement, (m) thermal imaging; (*) *p* < 0.05 indicates significant difference.

The lactylome results showed differences in lactylation of the ALDOA K230/322 loci in HCC cells and LCSCs. To validate this result, we used the ALDOA antibody to pull down ALDOA, and the anti‐Pan Kla antibody to detect the lactylation level of ALDOA. The level of ALDOA lactylation was significantly higher in LCSCs than in HCC cells (**Figure** **5**a). Treatment with 10 mm lactate significantly enhanced the level of lactylation (Figure 5b). In contrast, siLDHA treatment significantly inhibited the lactylation level of ALDOA in LCSCs compared to controls (Figure 5c). Similarly, treatment with glycolysis inhibitors 2‐DG (10 mm) and oxamate (20 mm) significantly reduced the lactylation level of ALDOA in LCSCs (Figure S6k,l, Supporting Information). To determine whether K230 and K322 are the primary lactylation sites of ALDOA, the site mutation assay was performed to mutate lysine K230/322 to arginine K230/322R in the human ALDOA gene, mimicking the de‐lactylation state of ALDOA (Figure S6m, Supporting Information). The results of the IP assay indicated a significant reduction in the lactylation level of ALDOA after the K230/322R mutation (Figure 5d). The ALDOA K230/322R mutation inhibited the proliferative capacity of LCSCs, as observed through CCK8, clone formation, and EdU proliferation staining assays (Figure 5e,f). The enzymatic activity of ALDOA and key indicators of glycolysis, such as glucose consumption, intracellular lactate production, and ATP production, were significantly reduced in LCSCs after ALDOA K230/322R mutation (Figure 5g). The extracellular flow detector Seahorse XF assay revealed that the glycolytic level and glycolytic capacity of LCSCs were significantly reduced after ALDOA K230/322R mutation (Figure 5h). Meanwhile, we found that the content of G6P, the upstream product of ALDOA, in the glycolytic pathway was significantly increased after the ALDOA K230/322R mutation in LCSCs, whereas the content of G3P, 2‐PG, and pyruvate, which are downstream product of ALDOA, was significantly decreased (Figure S7a, Supporting Information).

Next, the effect of ALDOA K230/322 mutation on the tumorigenic ability of LCSCs was detected by in vivo nude mice subcutaneous tumor formation assay. The same number of 3BL cells (GFP labeled) treated with ALDOA WT or ALDOA K230/322R were injected subcutaneously into the axilla of nude mice. 18 days later, tumor size was assessed by fluorescence imaging (Figure 5i). The results showed that ALDOA K230/322R‐treated LCSCs generated significantly lower tumor volume and weight compared to the ALDOA WT group (Figure 5j–l). Tumor tissues were then subjected to H&E and Ki67 immunohistochemistry experiments, which showed that ALDOA K230/322R treatment significantly reduced the number of proliferating cells in tumors compared to ALDOA WT‐treated LCSCs (Figure S7b, Supporting Information). The orthotopic implantation tumor model also showed smaller HCC tumor models formed by LCSCs that underwent the ALDOA K230/322R mutation compared to the WT group (Figure S7c, Supporting Information). The immunoprecipitation test results showed that the lactylation level of ALDOA in the orthotopic transplanted tumor formed by K230/322R mutation was significantly reduced, consistent with the results of cell experiments (Figure S7d, Supporting Information). To verify the changes in glycolysis ability of LCSCs in vivo after ALDOA K230/322R mutation, we used a thermal imager to detect the temperature at the tumor site in mice. The results showed that the temperature of the tumor formation site in nude mice with the ALDOA K230/322R mutation was significantly lower than that of the WT group (Figure 5m; Figure S7e, Supporting Information). These results indicate that the ALDOA K230/322R mutation significantly inhibits the proliferation ability and glycolysis level of LCSCs.

### 2.6 ALDOA Lactylation Promotes Stemness in LCSCs by Facilitating the Entry of DDX17 into the Nucleus

**Figure 6:**
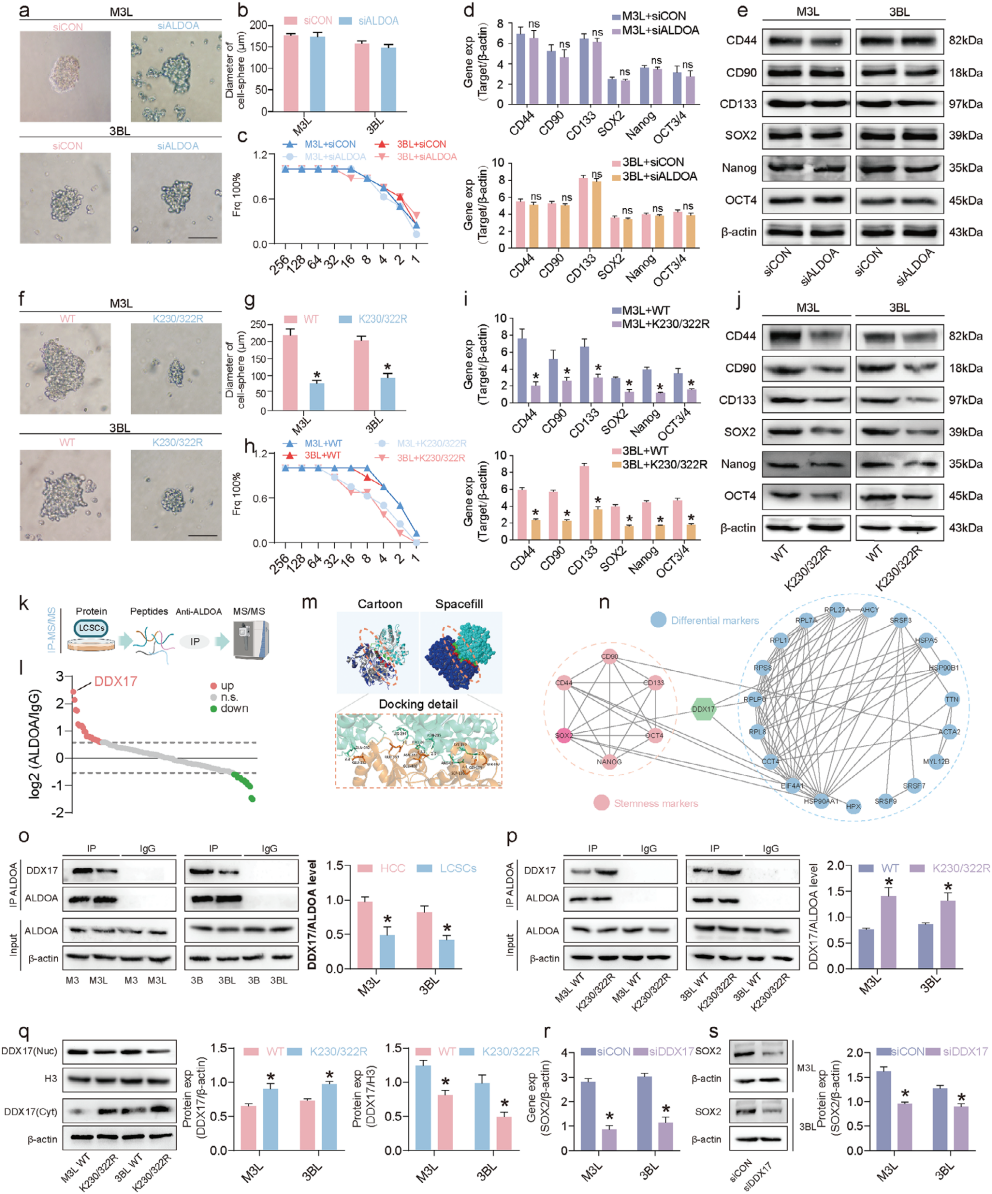
ALDOA lactylation enhances the regulatory function of DDX17 in maintaining the stemness of LCSCs. a,b) Sphere formation abilities of M3L and 3BL with siALDOA treatment (scale bar = 200 µm); c) M3L and 3BL frequency with siALDOA treatment were determined using in vitro LDA; d) qRT‐PCR detection of CSC marker‐related gene expressions with siALDOA treatment; e) Western Blotting detection of CSC marker‐related protein expressions with siALDOA treatment; f,g) Sphere formation abilities of M3L and 3BL with ALDOA K230/322R mutation treatment (scale bar = 200 µm); h) M3L and 3BL frequency with ALDOA K230/322R mutation treatment were determined using in vitro LDA; i) qRT‐PCR detection of CSC marker‐related genes expressions with ALDOA K230/322R mutation treatment; j) Western Blotting detection of CSC marker‐related protein expressions with ALDOA K230/322R mutation treatment; k)The flowchart of IP‐MS; l) Potential binding proteins for ALDOA; m) Molecular docking of ALDOA and DDX17; n) The PPI network between ALDOA's potential binding proteins and CSC marker related proteins; o) IP validation of ALDOA and DDX17 binding differences between HCC cells and LCSCs; p) The detection of ALDOA and DDX17 binding with IP in M3L and 3BL with ALDOA K230/322R mutation treatment; q) The detection of changes in DDX17 expression in the nucleus and cytoplasm of cells in M3L and 3BL with ALDOA K230/322R mutation treatment; r) qRT‐PCR detection of SOX2 gene expression with siDDX17 treatment; s) Western Blotting detection of SOX2 protein expression with siDDX17 treatment. (*) *p* < 0.05 indicates significant difference.

Next to investigate the effect of lactylation on LCSCs stemness, we first looked at the effect of the ALDOA protein itself on the stemness of LCSCs. The effect of siALDOA treatment on the size of LCSCs tumor spheres was first observed, and the results showed that there was no significant difference in the size of tumor cell spheres formed by LCSCs after siALDOA treatment (**Figure** **6**a,b). Then the effect of siALDOA on the stem cell frequency of LCSCs was observed by Limiting dilution analysis (LDA), and the results showed that siALDOA treatment had no significant effect on the stem cell frequency of LCSCs (Figure 6c). Meanwhile, the results of qRT‐PCR and Western Blotting experiments showed that siALDOA treatment had no significant effect on the mRNA and protein expression levels of CD44, CD90, CD133, SOX2, Nanog, and OCT4 in LCSCs (Figure 6d,e). These results above indicated that siALDOA had no significant effect on the stemness of LCSCs. Next, the exploration of the effect of ALDOA K230/322R mutation on the stemness of LCSCs was continued. The results showed a significant reduction in the diameter of LCSCs cell spheroids after 8 days of treatment with the ALDOA K230/322 mutation (Figure 6f,g). The LDA assay showed that the stemness frequency of M3L was reduced from 1:2.9 (95% confidence interval was 1:1.7–1:4.7) to 1:10.3 (95% confidence interval was 1:6.5–1:16.3) after K230/322R treatment, the stem cell frequency of 3BL was reduced from 1:3.4 (95% confidence interval was 1:2.2–1:5.5) to 1:12.8 (95% confidence interval was 1:8.2–1:20.2) (Figure 6h; Figure S7f,g, Supporting Information). The results of qRT‐PCR and Western Blotting experiments showed that ALDOA K230/322 mutation treatment significantly suppressed the mRNA and protein expression level of CD44, CD90, CD133, SOX2, Nanog, and OCT4 in LCSCs (Figure 6i,j). These results suggest that ALDOA undergoes lactylation that promotes the stemness of LCSCs, rather than the ALDOA protein itself.

To investigate the molecular mechanism by which ALDOA lactylation promotes stemness in LCSCs, we screened potential proteins interacting with ALDOA by Immunoprecipitation‐Mass Spectrometry (IP‐MS) (Figure 6k). DDX17, DEAD box deconjugate enzyme 17, which has been reported to be involved in the development and progression of several cancers, has been identified as a potential interacting protein (Figure 6l). The molecular docking analysis of ALDOA with DDX17 revealed that they bind with the lowest free energy (−20.5 kcal mol^−1^), indicating a strong binding activity between the two (Figure 6m). The protein‐protein interaction (PPI) network, which was generated by screening IP‐MS proteins and stemness marker proteins, revealed the binding of DDX17 to SOX2 (Figure 6n). Research has demonstrated that the nuclear expression of DDX17 amplifies its tumorigenic and stem cell‐like characteristics by facilitating the binding of SOX2 to its target genes.^[^ ^26^ ^]^ And our lactylome data shows that the lactylation of ALDOA mainly occurs in the cytoplasm (Figure 4e). IP results showed that ALDOA binding to DDX17 was significantly reduced in LCSCs compared to HCC cells (Figure 6o). Whereas, the ALDOA K230/322R mutation significantly promoted its binding to DDX17 (Figure 6p). Meanwhile, further analysis revealed that the expression of DDX17 in the nucleus of LCSCs was significantly reduced and the expression of DDX17 in the cytoplasm was significantly elevated after ALDOA K230/320R mutation (Figure 6q). Immunofluorescence results showed that, after the mutation of ALDOA K230/322 locus, the expression of ALDOA ontology did not change significantly, while the nuclear expression of DDX17 was significantly reduced, suggesting that lactylation of ALDOA K230/322 locus reduced its binding to DDX17 and thus promoted the entry of DDX17 into the nucleus of the cell, which was in line with the previous results (Figure S7h,i, Supporting Information). Finally, we verified the effect of DDX17 on the stemness of LCSCs and showed that siDDX17 suppressed the expression of stemness markers of LCSCs, including SOX2 (Figure 6r; Figure S7j,k, Supporting Information). These results indicate that lactylation of the ALDOA K230/322 site decreases it's binding to DDX17. This leads to the entry of DDX17 into the nucleus, which promotes the stem cell properties of LCSCs.

## 3 Discussion

HCC displays extremely inter‐tumor heterogeneity in terms of risk factors, clinical parameters, and molecular signatures, attributed to the existence of diverse cell clusters in HCC tissues.^[^ ^22^, ^27^ ^]^ Now, convincing evidence demonstrates that the HCC heterogeneity is fuelled by LCSCs, and these cells represent the root cause of tumor metastasis, recurrence as well as resistance to standard therapy.^[^ ^28^ ^]^ While numerous studies have investigated the molecular mechanisms underlying tumor progression mediated by cancer stem cells (CSCs), further research is needed to fully elucidate the function and associated mechanisms of their cellular metabolism. Enhanced glycolysis and lactate production play a crucial role in CSCs' self‐renewal and stemness. This study investigates how lactate maintains LCSCs' stemness and plasticity through lactylation, which regulates LCSCs' metabolism and tumorigenesis. These discoveries offer potential insights for the advancement of targeted therapy for LCSCs through the manipulation of the lactylation pathway.

Histone acylation marks comprise part of the hierarchy of epigenetic regulatory mechanisms. More recently, lactate‐derived histone lactylation was reported to directly regulate transcriptional processes of target genes from chromatin. Of note, multiple studies have indicated crosstalk between metabolic reprogramming and histone modifications. Many metabolites generated in metabolic pathways are known to serve as substrates for histone modifications. It is conceivable that enhanced glycolysis in HCC could result in lactate accumulation that could drive transcriptional alterations in genes related to malignant biological behavior of HCC in the extent of histone modifications. A recent study showed that higher glycolytic flux increase cellular lactylation levels, thereby enhancing pluripotency gene loci, opening them up to facilitate cellular reprogramming.^[^ ^29^ ^]^ Consistently, our results show that LCSCs display increased lactate production and histone lactylation levels, and that increasing H3K56 lactylation promote the expression of stemness related genes including OCT4.

The p300 is a crucial acyltransferase that catalyses a variety of protein modification types, such as acetylation, propanylation, butyylation, 2‐hydroxyisobutyration, and lactylation.^[^ ^30^, ^31^ ^]^ From a histone lactylation perspective, p300 has been discovered to participate in regulating inflammatory response and osteoblast differentiation.^[^ ^32^ ^]^ Defining the function of p300 in the LCSCs, we show that p300 expression was increased in the LCSCs, while the degree of H3K56 lactylation and OCT4 protein level decreased with p300 knockdown. Given that LDHA was reported to act as another important regulatory factor to lactylation, we set out to verify its regulatory function on H3K56 lactylation.^[^ ^33^ ^]^ In line with p300, we found that LDHA knockdown specifically reduced the degree of H3K56 lactylation and OCT4 protein expression in LCSCs. In addition, the LDHA knockdown inhibit the weight and volume of LCSCs‐induced HCC in vivo and prolonged the survival time of nude mice with transplanted tumors. Altogether, these results suggest that p300 and LDHA are the key manipulator in the reduction in H3K56 lactylation in LCSCs.

Furthermore, we identified lactylation residues by whole‐protein lactylation‐omics analysis and found that ALDOA K230/322 lactylation increased sharply in LCSCs, which was also involved in maintaining its stemness. ALDOA is a crucial glycolytic enzyme that is often aberrantly expressed in cancer cells, contributing to the Warburg effect. The large‐scale multi‐omics analysis of liver tissues revealed that ALDOA is elevated in HCC samples and this elevation is associated with the tumor progression and poorer patient overall survival. In line with this, we found that ALDOA is prominently increased in LCSCs, which apparently contributes to enhanced glycolysis and histone lactylation in LCSCs via a positive feedback loop.

Interestingly, ALDOA K230/322 lactylation regulates the separation of ALDOA and DDX17, which promotes the DDX17 entry into the nucleus, and in turn aggravate HCC. DDX17, a DEAD‐box RNA helicase, is a nuclear and cytoplasmic shuttle protein, which participates in modulating the physiological and pathological functions. DDX17 participate in modulating RNA metabolism processes, including the mRNA alternative splicing and degradation, and the coregulation of transcriptional activity; their abnormal expression is closely related to tumorigenesis and tumor progression. Moreover, previous studies indicated that DDX17 enhances stem‐like features by promoting its binding to its target genes. Indeed, our results revealed that lactylation/delactylation of ALDOA K230/322 determines the binding/separation of ALDOA and DDX17, and in turn regulate the stemness of LCSCs. This is also the first study to examine protein lactylation in the functions of proliferationand stemness characteristics of LCSCs.

In summary, our work reveals the surprisingly high contribution of lactylation to stemness maintenance of LCSCs, the key role played by lactate‐derived H3 histone and ALDOA K230/322 lactylation in which initiates stemness related gene transcription. Furthermore, LDHA inhibitors down‐regulate H3 histone and ALDOA K230/322 lactylation and alleviate the tumorigenic ability of LCSCs. Our study reveals that lactate directly modulates the malignant biological behaviors such as proliferation, migration, and stemness characteristics of LCSCs through lactylation, which could serve as a novel therapeutic strategy for HCC.

## 4 Experimental Section

### Cell Culture and Reagents

The human hepatocellular carcinoma cell lines HCCLM3 and Hep3B were provided by the Cancer Research Institute of Zhongshan Hospital, Fudan University. The cells were cultured at 37 °C with 5% CO_2_ and supplemented with 10% fetal bovine serum (FBS). Monolayer culture was maintained in high‐glucose medium with Gibco, Pleasanton, CA, USA, and treated with penicillin (100 U ml^−1^) and streptomycin (100 µg ml^−1^). LCSCs were enriched and identified from HCCLM3 and Hep3B using a previously described method.^[^ ^34^ ^]^ HCCLM3 LCSCs (M3L) and Hep3B LCSCs (3BL) were cultured in DMEM/F‐12 medium without vitamin A, supplemented with 20 ng ml^−1^ epidermal growth factor (EGF), 10 ng ml^−1^ basic fibroblast growth factor (FGF), 1% N‐2 and B‐27 (Life Technologies, Carlsbad, CA, USA). The medium was changed every other day. Experiments were conducted using M3L and 3BL above the P3 generation.

### Clone Formation Assay

HCCLM3, Hep3B, and P3 generation suspended LCSCs were extracted. Five hundred cells were added to each well of a 6‐well plate that had been cross‐linked with Matrigel 2 h in advance. After 5 days of inoculation, replace the old culture medium and continue culturing for another 5 days. Following the culture, gently wash the cells twice with PBS buffer, fix them with 4% paraformaldehyde for 4 h, and then stain them with crystal violet dyeing solution for 4 min. After staining, wash the cells twice with ddH_2_O and let the remaining water dry at room temperature. Finally, take photographs and count the number of cell colonies.

### Flow Cytometry

To determine the cell cycle, HCCLM3, Hep3B, and enriched P3 LCSCs were extracted. The cells were digested with trypsin and fixed overnight with 75% ethanol pre‐cooled at 4 °C. After incubation with PI staining solution at room temperature for 15 min away from light, the data was analyzed using FACS (Beckman, GER) and Cyto Expert software. To detect stemness markers of LCSCs, the cells were collected, digested with trypsin, and then incubated with phycoerythrin‐coupled anti‐CD44‐FITC (Becton Dickinson Labware, Bedford, MA, USA), CD90‐FITC resistant (Becton Dickinson Labware, Bedford, MA, USA), or CD133/FITC resistant (Miltenyi Biotec, Bergisch Gladbach, Westphalia Land, Germany). An appropriate isotype control was used for each antibody. The cells were then suspended in PBS. The data were analyzed using FACS (Beckman, GER) and Cyto Expert software.

### Real Time RT‐PCR and RNA Sequencing

Cells, whether treated or untreated, were collected at 0 °C and their total RNA was harvested using Trizol reagent, following a previously established approach.^[^ ^35^ ^]^ The RNA sequencing samples were then sent to BGI (Shenzhen, China) for transcriptome sequencing and analysis. The differentially expressed genes were verified using quantitative real‐time PCR (qRT‐PCR), with the primers listed in Table S1 (Supporting Information). β‐actin was selected as the internal control for normalization.

### Protein Extraction and Western Blotting

Treated and untreated cells were collected and lysed using RIPA protein extraction reagent (Beyotime, Beijing, China). The protein concentration was determined using the BCA protein assay kit (Beyotime, Beijing, China). Equal amounts of protein were loaded onto a 10% SDS‐PAGE gel and transferred to a PVDF membrane overnight. The membrane was then blocked with 5% skim milk powder for 1 h before incubating with the primary antibody (listed in Table S2, Supporting Information) at 4 °C overnight. Antibody information is presented in Table S2 (Supporting Information). The membrane was then washed and incubated with the secondary antibody connected to HRP at room temperature for 1 h. Finally, chemiluminescence (ECL, Thermo Scientific, USA) was used to observe samples from three separate experiments. Quantification was performed using Image J software.

### Extracellular Acidification and Oxygen Consumption Rate

An equal number of treated and untreated cells were inoculated into the Seahorse XF culture plate, with 6 × 10^4^ cells per well and 3 compound wells. The pore plates were cultured in a cell incubator for 24 h. Matrigel was used to cross‐link the orifice plates 2 h in advance. After the culture was complete, the culture plate was removed, and the cells were confirmed to be in good condition without contamination under the microscope. The stem cell culture medium was removed until only 100 µL remained per well. The cells were then washed three times with 500 µL of XF test solution and 500 µL of XF test solution was added. The culture plates were incubated in a CO_2_‐free cell incubator for 60 min. The pre‐hydrated test plate was then removed from the cell incubator and placed on the Seahorse work table. Successively, 10 mm glucose, 1 µm oligomycin, and 50 mm 2‐DG were added to the cell culture plate. The experiment was conducted by performing tests every 5 min in the cell incubator for a total of 100 min. The data obtained were then analyzed using the Agilent Seahorse Wave software.

### Metabolite Extraction and Gas Chromatography Mass Spectrometric (GC‐MS) Analysis

The experiment collected an equal number of treated and untreated cells, with more than 1 × 10^6^ per group and 8 replicates. The cells were then transferred to 1.5 mL EP tubes and washed three times with PBS solution. After discarding the supernatant, the cells were rinsed with 1 mL of 0.9% saline solution and centrifuged at 10 000 rpm for 5 min. The supernatant was then aspirated and discarded. The cells were quenched by freezing them in liquid nitrogen and thawing them at room temperature. This process was repeated three times. The centrifuge was then spun at 10 000 rpm for 5 min at 4 °C. The resulting supernatant was aspirated and added to a 1.5 mL EP tube. The sample was then dried using a nitrogen blower at 35 °C. Next, 80 µL of 20 ng ml^−1^ methoxy‐pyridine solution was added to the dried EP tube and incubated for 3 h at 37 °C. Fifty microliters of BSTFA was added to the sample, which was then vortexed, centrifuged, and incubated in a water bath at 80 °C for 1 h. After cooling at room temperature for 10 min and vertexing for 30 s, the supernatant was collected by centrifugation, stored at −20 °C, and tested within 48 h.^[^ ^36^ ^]^

### RNA Interference Experiments

The siRNAs targeting LDHA, p300, ALDOA, and DDX17, as well as negative controls, were designed and synthesized by RiboBio (Guangzhou, China). Following the manufacturer's protocol, Lipofectamine 3000 (Thermo Fisher, USA) was used to transfect cells. Cells were collected for RNA and protein level validation, as well as cell function testing. The siRNA oligo sequences are shown in Table S3 (Supporting Information).

### EdU Assay

The experiment involved seeding an equal number of cells in 24‐well plates at a density of 5 × 10^4^ cells per well. The plates were cross‐linked with matrigel 2 h prior to the experiment. After 12 h of culture, the medium containing 1×EdU working solution was replaced. The cells were then fixed using 4% PFA fixative and washed with PBS. Finally, the cells were treated with PBS pervious solution with Triton X‐100. The Click reaction solution was added and incubated at room temperature in the dark for 30 min. Then, Hoechst 33 342 solution was added and incubated at room temperature in the dark for 10 min. After the incubation period, the cells were washed with PBS. Finally, the cells were observed by fluorescence microscope, photographed, and the EdU positive rate was calculated as the ratio of EdU fluorescent cells to total cells in the same field of view.

### Spheroid Formation Assay and In Vitro Limited Dilution Analysis (LDA)

The stem cell medium was changed every other day, and after 15 days of culture, resulting spheres were photographed under a microscope. For in vitro LDA, LCSCs were seeded in 96‐well low adhesion culture plates at densities of 256, 128, 64, 32, 16, 8, 4, 2, and 1 per well, with 8 wells in each group. After 15 days of culture, the cells were observed under a microscope to determine if they formed spheres. The number of microspheres was recorded and the frequency of stem cells was calculated using the ELDA online website (https://bioinf.wehi.edu.au/software/elda/).

### ChIP Assay

LCSCs, treated or untreated, were harvested and used for ChIP assays. A chromatin immunoprecipitation kit (Beyotime, China) was used for the ChIP assay. Briefly, cells were fixed with 1% formaldehyde for 10 min, and the fixation reaction was quenched with glycine to a final concentration of 125 mm. Cells were lysed and sonicated until the desired length (100‐500 bp) was reached. Then, immunoprecipitation was performed with 5 µg of anti‐H3K56la or control IgG (Beyotime, China). Following the elution of DNA from the precipitated immune complexes, qRT PCR was conducted using the specific primers outlined in Table S4 (Supporting Information).

### Immunofluorescence

The human HCC tissue and normal paracancerous tissue in paraffin embedded sections were deparaffinized, rehydrated, fixed, and blocked using 5% bovine serum protein. The tissue sections were then incubated overnight with Pan Kla, anti‐CD44, and anti‐OCT4 antibodies at 4 °C. Subsequently, the slides were incubated with an HRP‐labeled secondary antibody for 30 min, and the cell nucleus was stained with DAPI (Sigma Aldrich). Digital images were obtained using an upright microscope (Leica, Germany) and fluorescence intensity was calculated. In the case of siLDHA treated nude mouse transplanted tumors, co‐transfection with anti‐H3K56la and OCT4 was performed. The clinical samples of HCC were purchased from Shanghai Outdo Biotech Company (Ethics Certificate: YB M‐05‐02).

### Cell Proliferation Assays

Equal numbers of treated and untreated LCSCs were seeded into low‐adhesion 6‐well plates and cultured for 12 h. Afterward, 100 µL of culture medium was aspirated from each well and transferred to a 96‐well plate. Then, 10 µL of CCK8 solution was added and transferred to a cell incubator for another 2 h of culture. At the end of the incubation, the 96‐well plates were removed, and the absorbance values were measured using a microplate reader set at 450 nm to calculate cell activity. The absorbance values were measured at 12, 24, 36, 48, 60, and 72 h, and a growth curve was plotted based on these values.

### Animal Experiment

Female nude mice (aged 6 weeks) were used in the experiments. The experimental protocol was approved and licensed by the Animal Ethics Committee of the Hubei University of Chinese Medicine (approval: 20220920046). Equal numbers of cells (1 × 10^6^ cells) from different treatment groups were inoculated in 100 µL of PBS and subcutaneously injected on both sides of nude mice at a 1:1 ratio. The longest axis (L) and vertical axis (R) of the tumor were measured daily after 7 days, along with the body weight of the mice. Tumor volume was measured every 2 days using the formula (tumor volume = (π/6) × length × width^2^). After 18 days, the temperature of the tumor site in mice was observed using a thermal imaging device. Alternatively, euthanize the mice and collect the tumor for subsequent research.

### Lactylome and Proteome

The cultured HCCLM3 cells (more than 1 × 10^7^ cells) and HCCLM3‐enriched LCSCs were washed with PBS buffer and collected by centrifugation. PTM Biolabs Co., LTD (Hangzhou, China) performed HPLC‐MS/MS analysis of Kla and database analysis, as well as functional enrichment analysis of differentially modified proteins.

### Immunoprecipitation

Immunoprecipitation (IP) was performed following the manufacturer's instructions for Protein A+G Agarose (Beyotime, Shanghai, China). First, the cells, treated or untreated, were lysed using IP lysate. Then, the corresponding primary antibody or IgG of the same species was incubated overnight with the cell lysate at 4 °C to form an immune complex. Subsequently, the fully resuspended Protein A+G Agarose was incubated with a mixture of antigen samples and antibodies at 4 °C for 2 h and mixed. Finally, the binding protein was washed for Western blotting analysis.

### Glycolysis Index Detection

The ALDOA enzyme activity (Genmed, Shanghai, China), glucose content (Leagene, Anhui, China), ATP content (Beyotime, Shanghai, China), lactate content (Abbkine, USA), G6P content (Yansheng, Shanghai, China), G3P content (Yansheng, Shanghai, China), 2‐PG content (Weibo, Guangzhou, China), and pyruvate content (Solarbio, Beijing, China) were tested after lysing the cells, following the manufacturer's instructions for the reagent kit.

### Statistical Analysis

The data from three independent experiments were collected and represented as mean ± standard deviation (SD). For qRT‐PCR and western blotting, β‐actin was normalized for all significance analyses, including one‐way analysis of variance (ANOVA), multiple comparisons, and t‐tests, using Origin software. A value of *p* < 0.05 was considered statistically significant.

## Conflict of Interest

The authors declare no conflict of interest

## Author Contributions

F.F., J.W., and Q.C. contributed equally to this work. K.X. and C.W. conceived, designed, and supervised the experiments. L.P., F.F., S.W., and J.W. performed the experiment. G.S., W.L., K.X., and F.F. analyzed the data and prepared figures. K.X., C.W., and L.Y. wrote the manuscript. Q.C., K.X., and C.W. provided the financial supports. The authors declare that all data were generated in‐house and that no paper mill was used.

## Supporting information

Supporting Information

## Data Availability

The data that support the findings of this study are available in the supplementary material of this article.
