# Supplementary material for: Lactylome Analysis Unveils Lactylation‐Dependent Mechanisms of Stemness Remodeling in the Liver Cancer Stem Cells
Source: Adv Sci (Weinh). 2024 Aug 5;11(38):2405975. doi: 10.1002/advs.202405975 (PMC11481176; doi:10.1002/advs.202405975)
Supplement: Supplementary file 1 — Supporting Information [file ADVS-11-2405975-s001.docx]

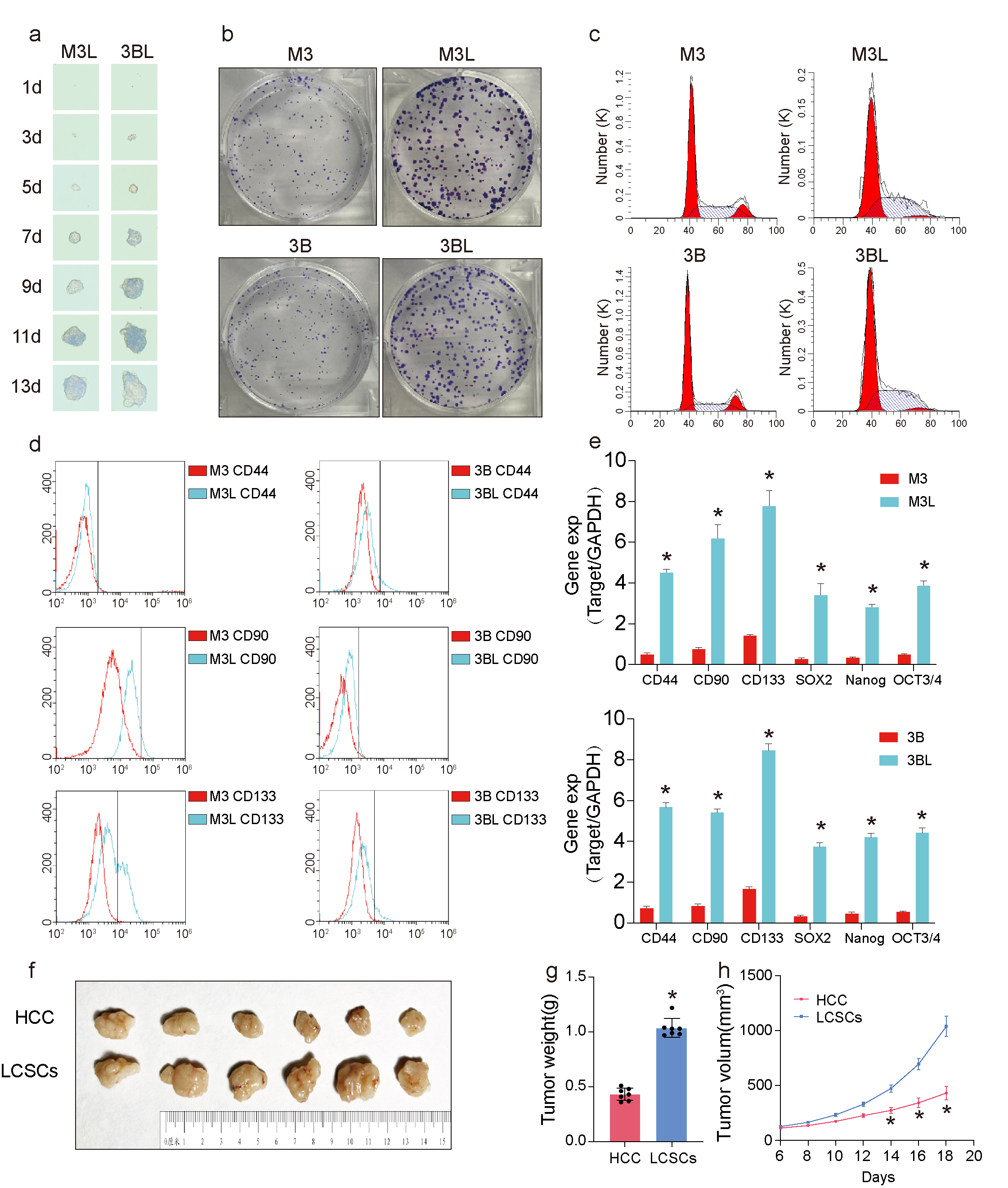


**Figure S1.** Enrichment and characterisation of LCSCs. (a) Process of LCSCs formation by LCSCs; (b) Clone formation ability of LCSCs versus HCC cells; (c) Comparison of flow cytometry cycle detection between HCC cells and LCSCs; (d) Expression of CD44, CD90 and CD133 in HCC cells and LCSCs was determined by flow cytometry; (e) qRT-PCR detection of CSC marker-related gene expressions; (f, g, h) *In vivo* tumor formation of LCSCs versus HCC cells, (g) Tumor weight measurement, (h) Tumor volume measurement. (*) p < 0.05 indicates significant difference.


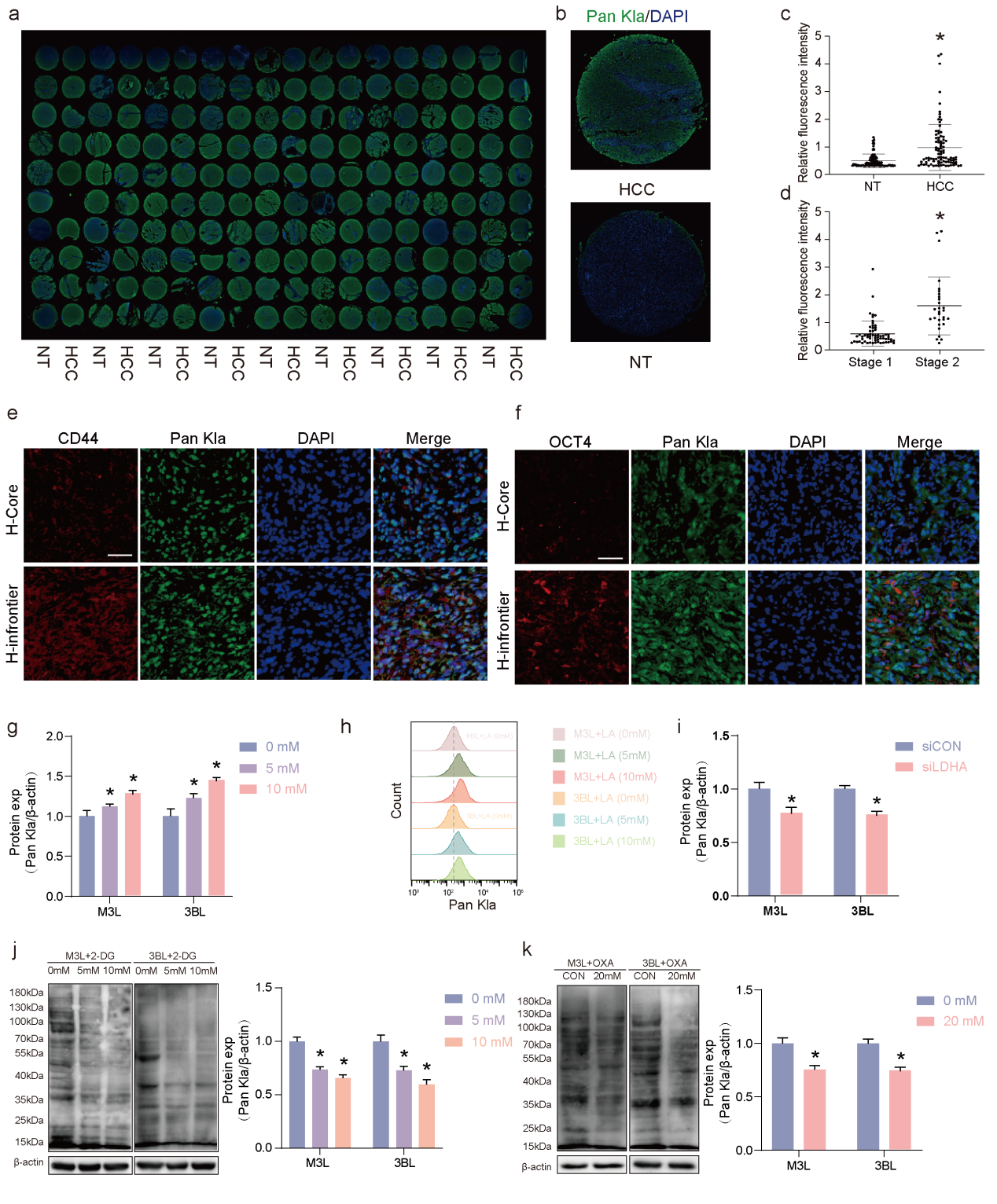


**Figure S2.** The level of lactylation in the invasive frontier region of HCC cancer tissue is significantly higher than that in the core region. (a, b) Pan Kla immunofluorescence staining of tissue chips from 90 HCCs and 90 adjacent tissues (n=90); (c, d) Quantitative analysis of Pan Kla expression in (A); (e, f) CD44 and OCT4 co stained with Pan Kla in the invasion frontier and core regions of HCC tissue (scale bar = 100 μm); (g) Quantitative analysis of Pan Kla expression in Figure 2e; (h) Expression of Pan Kla in LCSCs after exogenous lactate addition detected by flow cytometry; (i) Quantitative analysis of Pan Kla expression in Figure 2f; (j, k) The Pan-Kla detection of M3L and 3BL with 2-DG and OXA treatments. (*) p < 0.05 indicates significant difference.


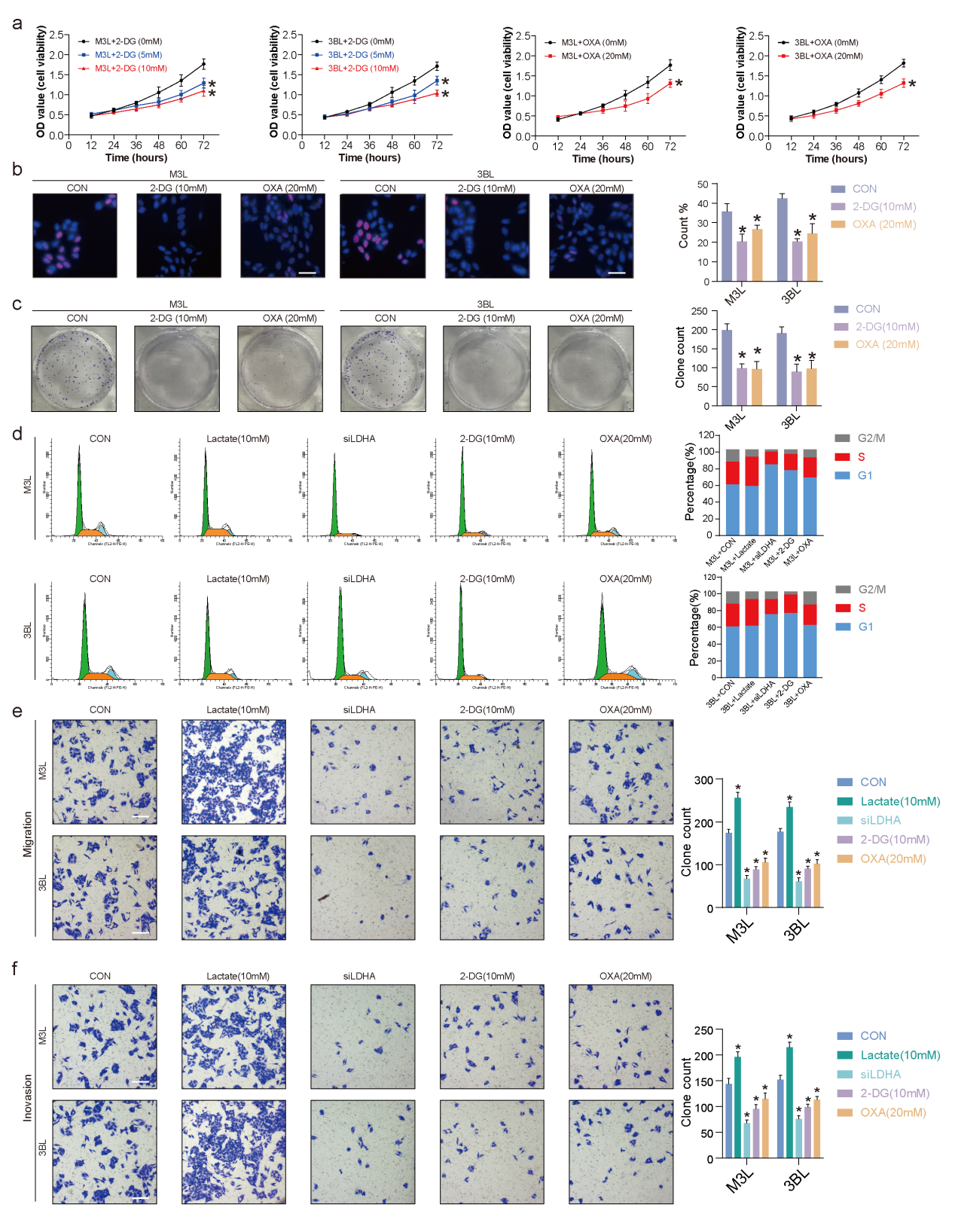


**Figure S3**. Effects of glycolysis inhibitors on the biological behavior of LCSCs. (a) The cell viabilities of M3L and 3BL with 2-DG and OXA treatments; (b) Cell proliferation from EdU assay of M3L and 3BL with 2-DG and OXA treatments (scale bar = 50 μm); (c) Colony formation abilities of M3L and 3BL with 2-DG and OXA treatments; (d) Cell cycle changes in LCSCs after treatment with lactate, siLDHA, 2-DG and OXA detected by flow cytometry; (e) Transwell detection of changes in migration of LCSCs after treatment with lactate, siLDHA, 2-DG, and OXA; (f) Transwell detection of changes in invasion of LCSCs after treatment with lactic acid, siLDHA, 2-DG, and OXA. (*) p < 0.05 indicates significant difference.


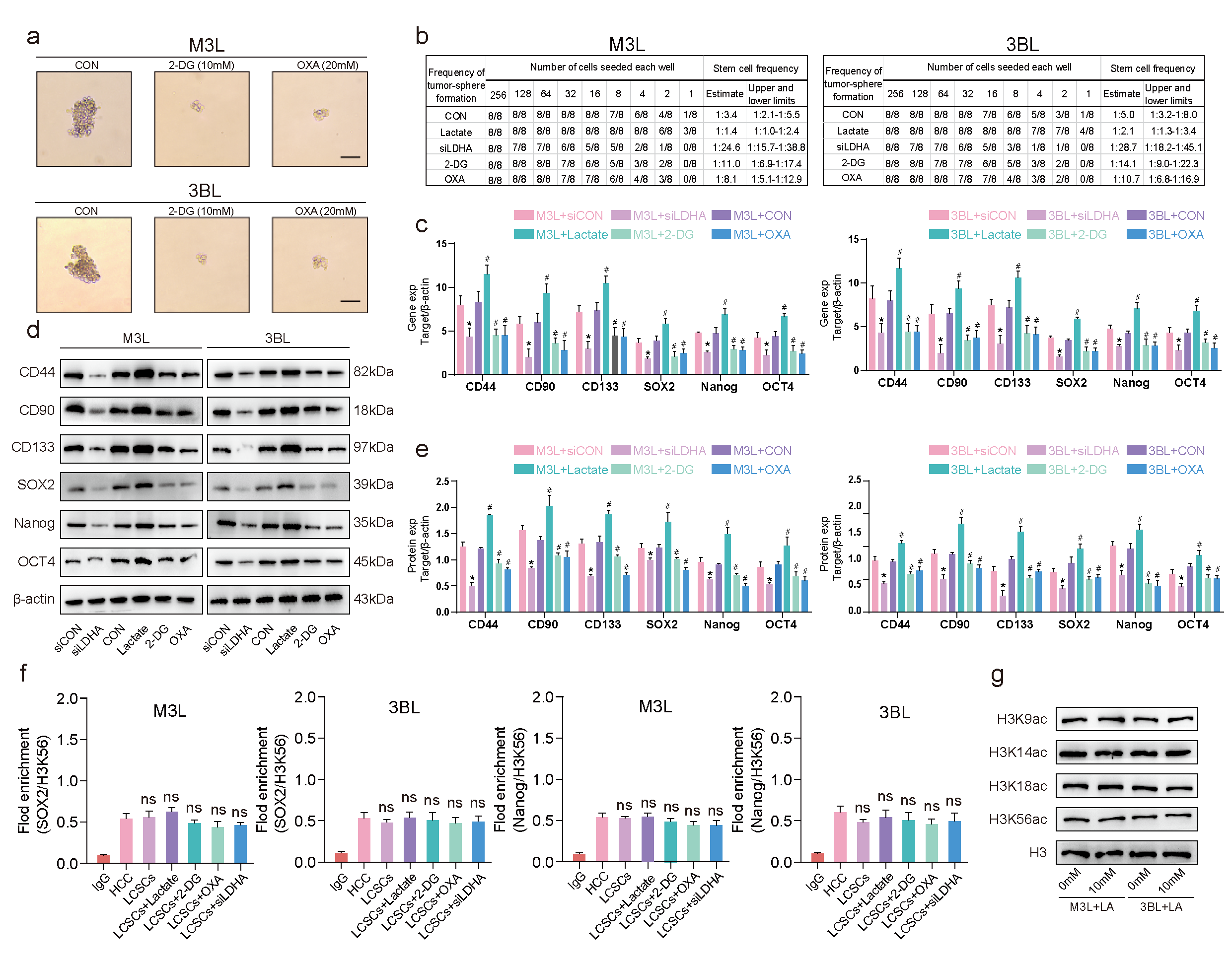


**Figure S4**. Effect of glycolysis inhibitors on the dryness of LCSCs. (a) Sphere formation abilities of M3L and 3BL with 2-DG and OXA treatments (scale bar = 100 μm); (b) M3L and 3BL frequency with 2-DG and OXA treatments were determined using *in vitro* LDA; (c) Effect of lactate, siLDHA, 2-DG, and OXA on mRNA expression of stemness markers in LCSCs by qPCR(^*^p < 0.05, compared to siCON) (^#^p < 0.05, compared to CON); (d, e) Effect of lactate, siLDHA, 2-DG, and OXA on protein expression of stemness markers in LCSCs by Western blotting; (f) ChIP-PCR analyses of SOX2 and Nanog via reacting with immunoprecipitation of H3K56la antibody in LCSCs with different treatments; (g) Effect of lactate on protein expression of H3K9ac, H3K14ac, H3K18ac and H3K56ac in LCSCs by Western blotting. (*) p < 0.05 indicates significant difference.


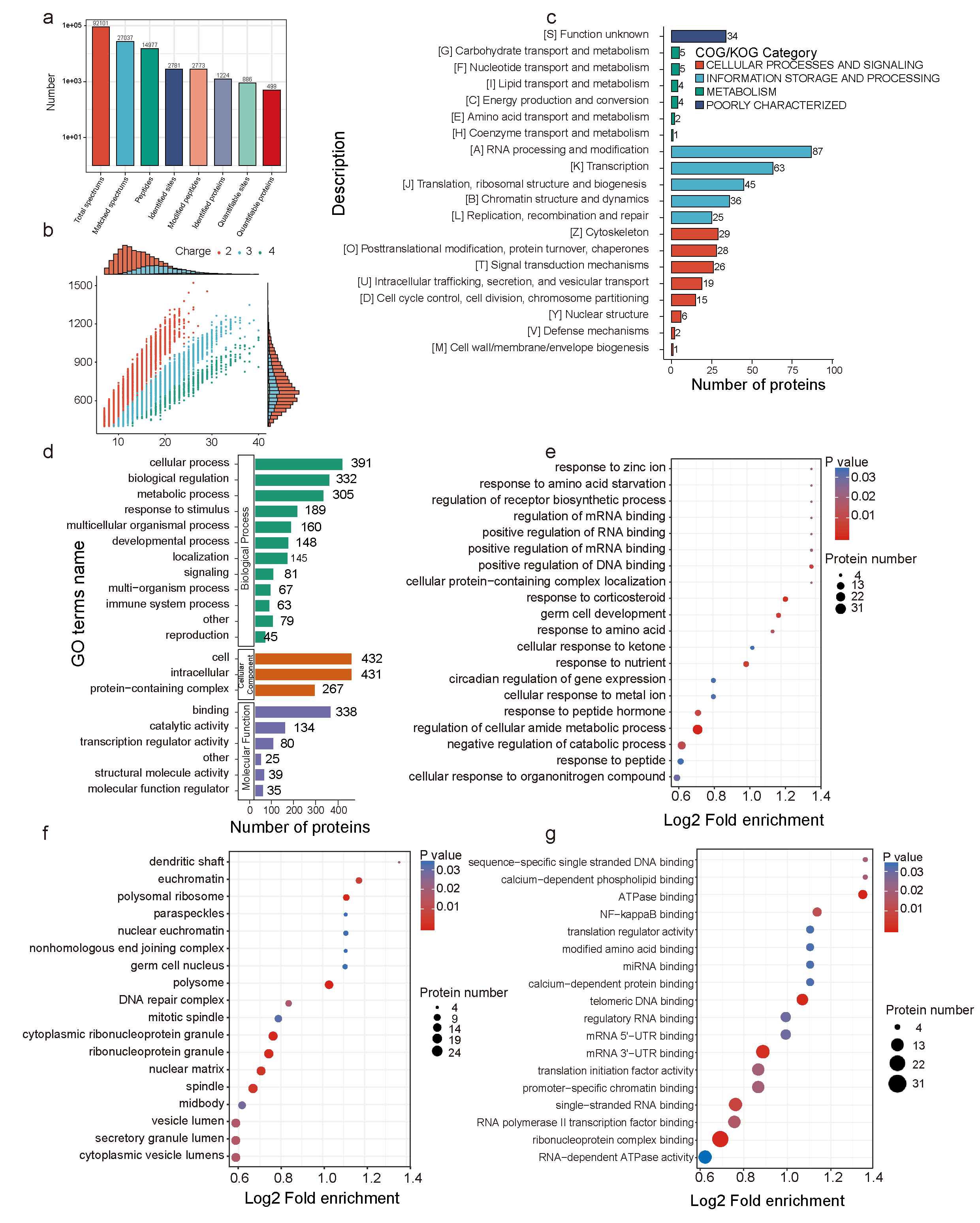


**Figure S5.** lactylome of M3 and M3L. (a, b) Lactylation omics quality control; (c) COG/KOG function of differential modified protein; (d) GO secondary classification; (e) GO cell components enrichment analysis; (f) GO biological processes enrichment analysis; (g) GO molecular functions enrichment analysis.


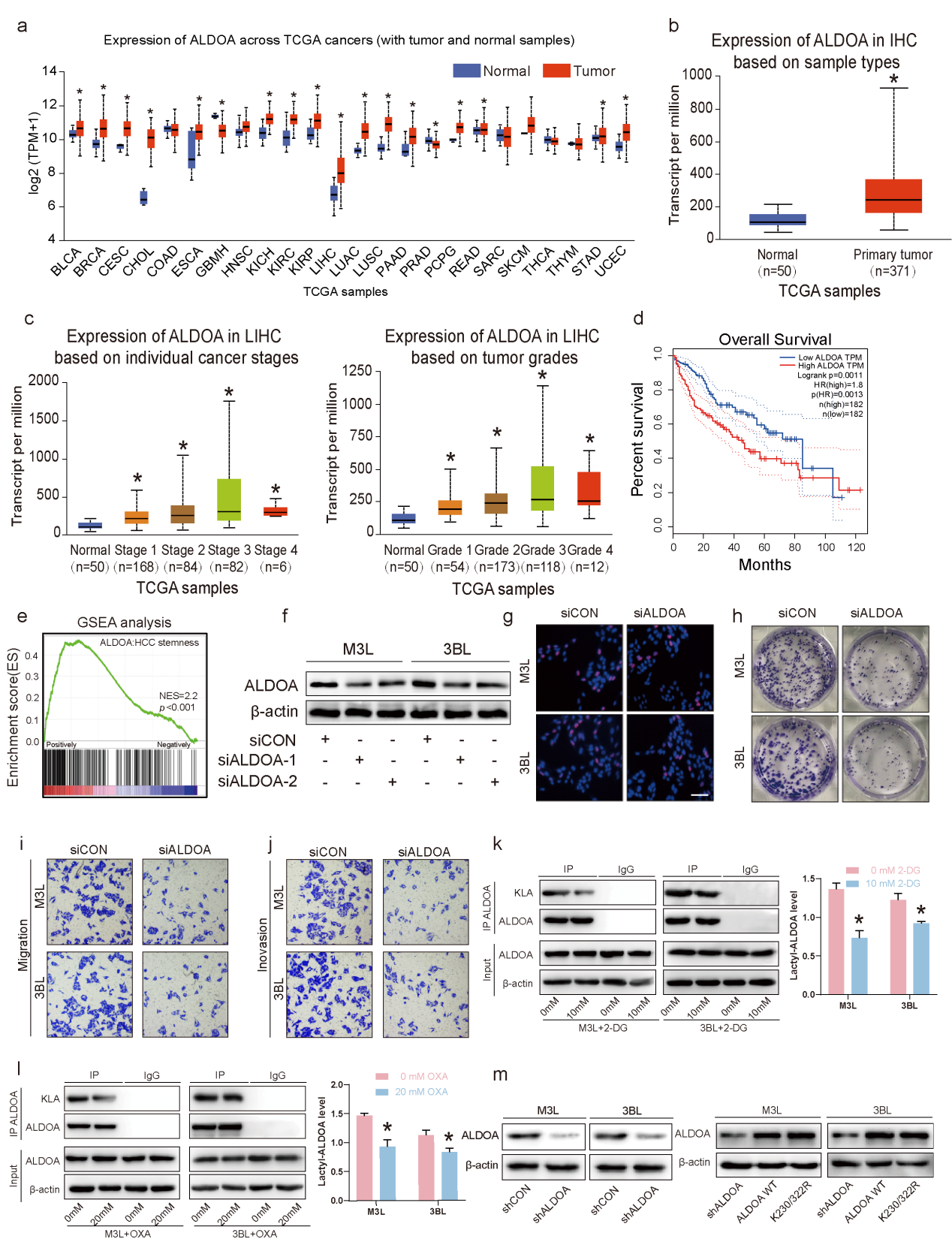


**Figure S6.** Bioinformatics analysis of ALDOA in HCC. (a) Expression differences of ALDOA in different tumor tissues; (b) ALDOA expression difference between HCC cancer and normal tissues; (c) The varying expression of ALDOA in different stages and grades of HCC; (d) K-M curve plot of the relationship between changes in ALDOA expression and survival rate and prognosis prediction in HCC patients; (e) Analysis of the correlation between ALDOA and HCC stemness; (f) Western blotting detection of ALDOA konckdown effect; (g) Cell proliferation from EdU assay of M3L and 3BL with siALDOA treatment (scale bar = 50 μm); (h) Colony formation abilities of M3L and 3BL with siALDOA treatment; (i) Transwell detection of changes in migration of LCSCs after siALDOA; (j) Transwell detection of changes in invasion of LCSCs after siALDOA; (k) The detection of ALDOA lactylation with IP in M3L and 3BL with 2-DG treatment; (l) The detection of ALDOA lactylation with IP in M3L and 3BL with OXA treatment; (m) Effect of ALDOA K230/322R mutation on ALDOA expression. (*) p < 0.05 indicates significant difference.


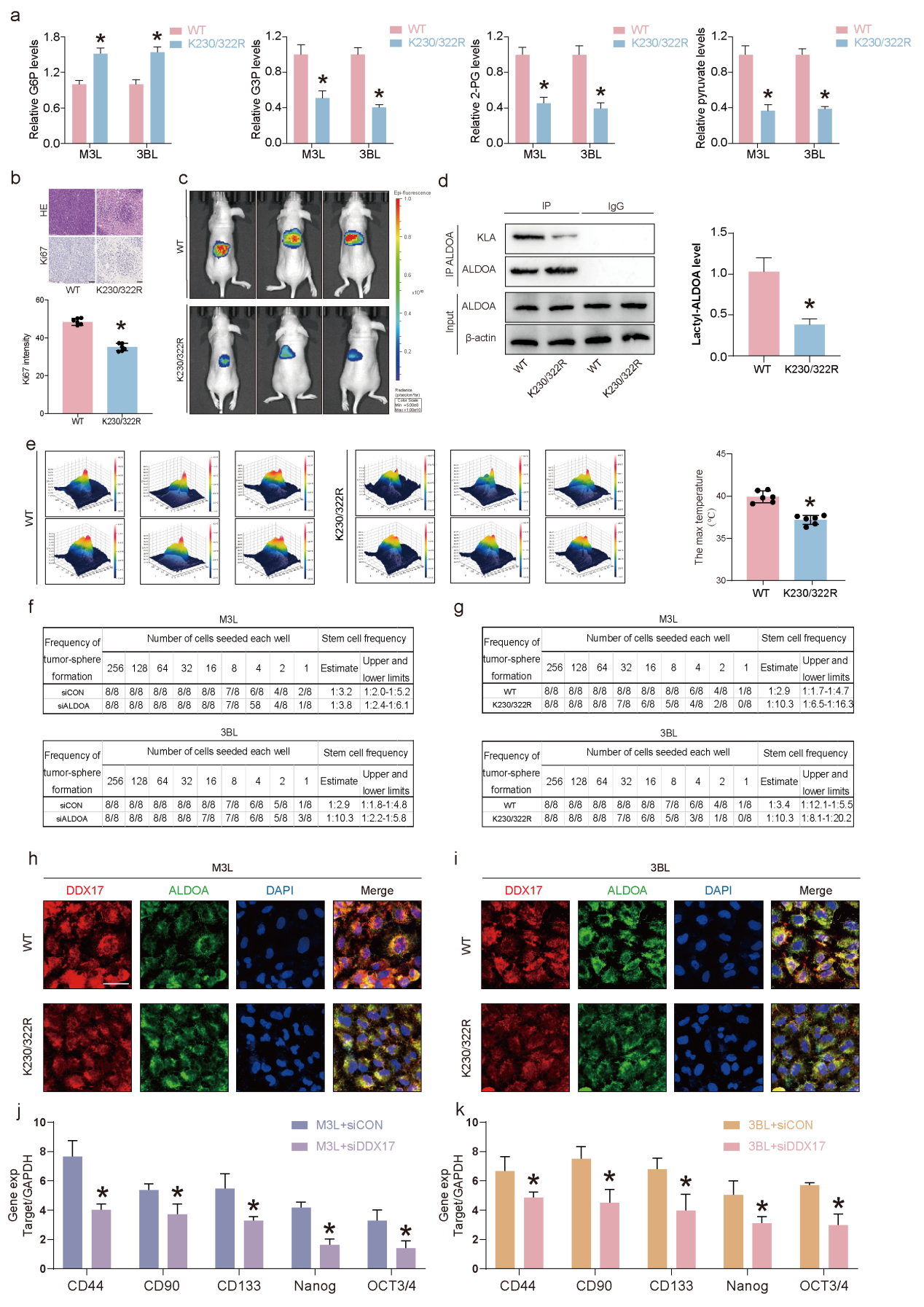


**Figure S7.** The effects of ALDOA K230/322R mutation on LCSCs. (a) G6P, G3P, 2-PG and pyruvate content detection; (b) Histological and immunostaining evaluation of LCSCs *in vivo* tumor tissues with ALDOA K230/322R mutation (scale bar = 100 μm); (c) The orthotopic implantation tumor model to detect the effect of the K230/322 mutation on the tumorigenic capacity of LCSCs; (d) Immunoprecipitation detection of changes in ALDOA lactylation levels in orthotopic implantation tumors; (e) Maximum temperature of tumors; (f) M3L and 3BL frequency with siALDOA treatment were determined using *in vitro* LDA; (g) M3L and 3BL frequency with ALDOA K230/322R mutation treatment were determined using *in vitro* LDA; (h, i) Immunofluorescence detection of DDX17 expression changes in M3L and 3BL after K230/322 mutation (scale bar = 20 μm); (j, k) qRT-PCR detection of CSC marker-related gene expressions with siDDX17 treatment. (*) p < 0.05 indicates significant difference.

Table S1. Primer sequences for real-time RT-PCR

| Gene | Sense |
| --- | --- |
| CD44 | 5′ CTGCCGCTTTGCAGGTGTA 3′ 5′ ATTGTGGGCAAGGTGCTATT 3′ |
| CD90 | 5′ CAGCATCGCTCTCCTGCT AA 3′ 5′ ACTGGATGGGTGAACTGCTG 3′ |
| CD133 | 5′ TGGAGCGTCCCTTCACCC 3′ 5′ TTTCTCAAAGTATCTGGATGTAGCA 3′ |
| SOX2 | 5′ AGACAGCTCTGTTCAGTATT 3′ 5′ TTACACCAGCCTCCAAGA 3′ |
| Nanog | 5′ CCCCAGCCTTTACTCTTCCTA 3′ 5′ CCAGGTTGAATTGTTCCAGGTC 3′ |
| OCT4 | 5′ TGAGTAGTCCCTTCGCAAGC 3′ 5′ TTAGCCAGGTCCGAGGATCA 3′ |
| β-actin | 5′ CTCCATCCTGGCCTCGCTGT 3′ 5′ GCTGTCACCTTCACCGTTCC 3′ |
| Glut1 | 5′ TCACTGTGCTCCTGGTTCTGTTC 3′ 5′ GCTCCTCGGGTGTCTTGTCA 3′ |
| HK1 | 5′CCAACATTCGTAAGGTCCATTCC3′ 5′CCTCGGACTCCATGTGAACATT 3′ |
| GPI | 5′ CAAGGACCGCTTCAACCACTT 3′ 5′ CCAGGATGGGTGTGTTTGACC 3′ |
| PFKP | 5′CGCCTACCTCAACGTGGTG 3′ 5′ ACCTCCAGAACGAAGGTCCTC 3′ |
| ALODA | 5′ GCTGTCACTGGGATCACCTTC 3′ 5′ GCTCGGAGTGTACTTTCCTTGA 3′ |
| PGK1 | 5′ GACCTAATGTCCAAAGCTGAGAA 3′5′ CAGCAGGTATGCCAGAAGCC 3′ |
| ENO1 | 5′ GCCGTGAACGAGAAGTCCTG 3′ 5′ ACGCCTGAAGAGACTCGGT 3′ |
| PKM2 | 5′ AAGGGTGTGAACCTTCCTGG 3′ 5′ GCTCGACCCCAAACTTCAGA 3′ |
| LDHA | 5′ GATTCAGCCCGATTCCGTTAC 3′ 5′ GAGTCCAATAGCCCAGGATGTG 3′ |
| DDX17 | 5′ GTGTTTGCCTTCCATCAT 3′ 5′ TCTTCCCAGAGCCAGTC 3′ |

Table S2. Antibody for Western Blotting

| Antibody | Cat No | Producer |
| --- | --- | --- |
| anti-Glut1 | R380464 | ZenBio, China |
| anti-HK1 | R24551 | ZenBio, China |
| anti-GPI | R383002 | ZenBio, China |
| anti-PFKP | R25341 | ZenBio, China |
| anti-ALDOA | 11217-1-AP | Proteintech, China |
| anti-PGK1 | R25343 | ZenBio, China |
| anti-ENO1 | R23329 | ZenBio, China |
| anti-PKM2 | R381318 | ZenBio, China |
| anti-LDHA | R24822 | ZenBio, China |
| anti-β-actin | R51031 | ZenBio, China |
| anti-Pan Kla | PTM-1425 | PTM BIO, China |
| anti-CD44 | R23842 | ZenBio, China |
| anti-CD133 | 252208 | ZenBio, China |
| anti-CD90 | R22754 | ZenBio, China |
| anti-SOX2 | R30004 | ZenBio, China |
| anti-Nanog | R381167 | ZenBio, China |
| anti-OCT4 | R381335 | ZenBio, China |
| anti-H3K9la | PTM-1419RM | PTM BIO, China |
| anti-H3K14la | PTM-1414RM | PTM BIO, China |
| anti-H3K18la | PTM-1406RM | PTM BIO, China |
| anti-H3K56la | PTM-1421RM | PTM BIO, China |
| anti-H4K5la | PTM-1407RM | PTM BIO, China |
| anti-H4K8la | PTM-1415RM | PTM BIO, China |
| anti-H4K12la | PTM-1411RM | PTM BIO, China |
| anti-H4K16la | PTM-1417RM | PTM BIO, China |
| anti-p300 | 347220 | ZenBio, China |
| anti-DDX17 | R389107 | ZenBio, China |
| anti-H3K9ac | PTM-112RM | PTM BIO, China |
| anti-H3K14ac | PTM-157 | PTM BIO, China |
| anti-H3K18ac | PTM-114RM | PTM BIO, China |
| anti-H3K56ac | PTM-162 | PTM BIO, China |

Table S3. RNA oligo for siRNA transfection

| Gene | Sense |
| --- | --- |
| siLDHA | 5′-GGCAAAGACTATAATGTAA-3′ |
| shLDHA | 5′-GGCAAAGACTATAATGTAA-3′ |
| sip300 | 5′-GGCAAAGACTATAATGTAA-3′ |
| siALDOA | 5′-GGCGUUGUGUGCUGAAGAUTT-3′ |
| siDDX17 | 5′-GCUGCUUAUGGCACCAGUAGCUAUA-3′ |
| siCON | 5′-GATCATACGTGCGATCAGA-3′ |

Table S4 ChIP-PCR primer sequences

| Genes | Primer Sequences (Forward/Reverse) |
| --- | --- |
| OCT4 | 5′-ACATGTGTAAGCTGCGGCC-3′  5′-GTTGTGCATAGTCGCTGCTT-3′ |
| SOX2 | 5′-CCAGTAGGCGATGCAAGTTA-3′  5′-CTAGACGCCCGCAACCTG-3′ |
| Nanog | 5′-CAAAGGCAAACAACCCACTT-3′  5′-CATCCCTGGTGGTAGGAAGA-3′ |
